# A combined genome-wide association and molecular study of age-related hearing loss in *H. sapiens*

**DOI:** 10.1186/s12916-021-02169-0

**Published:** 2021-12-01

**Authors:** Wei Liu, Åsa Johansson, Helge Rask-Andersen, Mathias Rask-Andersen

**Affiliations:** 1 Department of Surgical Sciences, Section of Otorhinolaryngology and Head & Neck Surgery, Uppsala University, SE-751 85 Uppsala, Sweden; 2 Department of Immunology, Genetics and Pathology, Science for Life Laboratory, Uppsala University, Uppsala, Sweden

**Keywords:** Age-related hearing loss, Human gene expression, Structured illumination microscopy, GWAS

## Abstract

**Background:**

Sensorineural hearing loss is one of the most common sensory deficiencies. However, the molecular contribution to age-related hearing loss is not fully elucidated.

**Methods:**

We performed genome-wide association studies (GWAS) for hearing loss-related traits in the UK Biobank (*N* = 362,396) and selected a high confidence set of ten hearing-associated gene products for staining in human cochlear samples: EYA4, LMX1A, PTK2/FAK, UBE3B, MMP2, SYNJ2, GRM5, TRIOBP, LMO-7, and NOX4.

**Results:**

All proteins were found to be expressed in human cochlear structures. Our findings illustrate cochlear structures that mediate mechano-electric transduction of auditory stimuli, neuronal conductance, and neuronal plasticity to be involved in age-related hearing loss.

**Conclusions:**

Our results suggest common genetic variation to influence structural resilience to damage as well as cochlear recovery after trauma, which protect against accumulated damage to cochlear structures and the development of hearing loss over time.

**Supplementary Information:**

The online version contains supplementary material available at 10.1186/s12916-021-02169-0.

## Background

In humans, hearing loss is a potentially debilitating condition that affects more than 1.23 billion people worldwide and constitutes one of the world’s top ten causes of years lived with disability [1]. The most common form of hearing loss, which represents 90% of all cases, is related to the degenerative effects of aging on hearing, i.e., age-related hearing loss or presbycusis. This is followed by inflammatory disease of the middle ear (otitis media) and congenital anomalies such as non-syndromic deafness due to genetic factors [1] as the most common causes. Age-related hearing loss is most commonly bilaterally symmetrical, progressive, and irreversible. No preventative therapy exists and symptomatic treatment usually involves the use of hearing aids or surgical implantation of cochlear implants.

The molecular mechanisms that underlie the development of hearing loss and individual variation in risk are poorly elucidated. Twin studies have demonstrated a moderate-to-high heritability for age-related hearing loss of 40–70% [2–6] and several genetic factors and genes have been linked to rare non-syndromic congenital hearing loss. However, the genetic factors that influence variation in risk, emergence, and progression of age-related hearing loss in the general population are poorly characterized.

Over the last decade, genome-wide association studies (GWAS) have been able to illuminate the genetic architectures behind a number of human traits and diseases. Recently, genetic data have become available from large cohorts with standardized collection of many phenotypic traits. For example, in the UK Biobank, a cross-sectional cohort of more than half a million United Kingdom (UK) residents, information on hearing-related traits was collected by touchscreen questionnaire as well as speech-in-noise hearing tests. A recent GWAS of self-reported hearing difficulty and hearing aid use in ~ 250,000 UK Biobank participants identified 44 genetic loci that were associated with these traits and were able to demonstrate the utility of self-reported data for identification of hearing loss-associated genetic variants. In addition, immunological staining revealed murine cochlear expression of three novel hearing-associated proteins: nidogen-2 (encoded by *NID2*), clarin-2 (*CLRN2*), and the Rho guanine nucleotide exchange factor 28 (*ARHGEF28*) [7].

The cochlea is the critical organ for the sense of hearing and several structural and metabolic components of the human cochlea have been linked to rare forms of syndromic and non-syndromic deafness, such as the gap-junction protein connexin 26 (encoded by *GJB2*) [8] and prestin (*SLC26A5*), the motor protein of the outer hair cells [9]. Imaging of these components in cochlear tissue samples has provided substantial insights into their role in hearing. By necessity, imaging studies have defaulted to laboratory animals due to the paucity of human samples. However, differences in anatomy and molecular function of cochlear components between mammals can complicate extrapolation of results to humans.

In this study, we visualize human hearing-associated proteins, identified as candidates by GWAS, in human cochlear samples by immunohistochemical staining combined with super-resolution structured illumination microscopy (SR-SIM). Human cochlear samples were collected during surgical removal of life-threatening petroclival meningioma and directly fixed for optimal preservation and antigenicity of protein structures.

## Results

### Genome-wide association analyses

Cohort characteristics and summaries of the phenotypic data are presented in Additional file 1: Table 1 [10–115]. GWAS of hearing-related phenotypes in the UK Biobank revealed 67 loci that were associated with at least one hearing-related phenotype (*P* < 5 × 10^−8^): 35 with hearing difficulty, 22 with hearing aid use, eleven with tinnitus, and two with speech-in-noise (Additional file 1: Fig. 1-2, Additional file 2). SNP heritability estimates ranged from 0.94% for hearing aid use to 3.45% for hearing difficulty (Additional file 1: Table 2). There were no indications of genomic inflation for all GWAS except for tinnitus, which indicates that genomic inflation due to stratification may be present in this dataset (Additional file 1: Table 2). After adjusting for genomic inflation, no associations remained for tinnitus.

Seven loci were associated with both hearing difficulty and hearing aid use, while one locus was associated with both hearing difficulty and speech-in-noise. Associations were observed at six previously described autosomal non-syndromal hearing loss loci: DFNA10 (deafness autosomal dominant 10), which contains the gene *EYA4* (eyes absent homolog 4); DFNB28 (deafness autosomal recessive 28), which contains *TRIOBP* (TRIO and F-actin-binding protein); DFNB61, which contains the gene that encodes prestin, *SLC26A5*; DFNA58, DFNB47, and DFNB79, which contains *TPRN* (taperin) and *CACNA1B* (voltage-dependent N-type calcium channel subunit alpha-1B, Additional file 1: Table 3). A review of the literature revealed that 26 out of 67 hearing associated loci contain genes that have been linked to hearing or hearing-related traits in published peer-reviewed original research (Additional file: Table 3). Seven loci in our GWAS where the lead SNP was just below our threshold for significance (*P* < 5e−7), also overlapped with additional non-syndromal deafness-associated loci: DNFB36, which contains *ESPN* (espin), DFNB42, which contains *ILDR1* (immunoglobulin-like domain-containing receptor 1), DFNB12, which contains *CDH23* (cadherin 23), DFNA3A, which contains *GJB2* (connexin26), DFNA9, which contains *COCH* (cochlin), DFNA4B, which contains *CEACAM16* (carcinoembryonic antigen-related cell adhesion molecule 16), and DFNB8/10, which contains *TMPRSS3* (Transmembrane protease serine 3, Additional file 2).

Overlaps with missense coding SNPs were found at 15 loci and potentially deleterious variants were found at seven of these loci, within *CCR9*, *FSCN2*, *TYR*, *SYNJ2*, *SLC16A8*, *KLHDC7B*, and *HLA-DQB1* (Additional file 1: Table 4). Overlap with expression quantitative trait loci (eQTLs) was also found at seven loci (Additional file 1: Table 5). However, these eQTLs were not found to regulate any genes that have previously been linked to hearing-related traits in the literature. It should be mentioned that the GTEx database presently does not contain data on gene expression in components of the human ear, which may limit our ability to identify eQTLs that can be linked to hearing or hearing-related traits.

Due to the rare opportunities to collect human cochlear tissue during surgery for staining, and thereby the limited amount of tissue for staining, the genes selected for functional analyses were selected with care. A total of ten genes were prioritized based on review of the literature on associated genes as well as our bioinformatic analyses. Accordingly, a “high-confidence” set of candidate genes was generated (Table 1), which contained genes that have all been linked to hearing-related phenotypes in humans or other species. Candidate protein expression patterns in other mammals are summarized in Additional file 1: Table 6.

**Table 1:** Candidate genes and rationale for selection for staining in human cochlear samples. Entries are sorted by ascending *p* values

Candidate gene	Protein name	Associated phenotype	Selection rationale	Lead SNP	Chr	Coordinates (bp)	P
*TRIOBP*	TRIO and F-actin-binding protein	Hearing difficulty	dbSNP, literature	rs739138	22	38,116,507–38,157,805	4.08E−12
*PTK2/FAK*	Focal adhesion kinase 1	Hearing difficulty	Proximity, literature	rs1962104	8	141,604,684–141,932,130	6.25E−11
*UBE3B*	Ubiquitin-protein ligase E3B	Hearing difficulty	dbSNP, literature	rs1558804	12	109,788,598–110,042,348	1.57E−10
*MMAB*	Corrinoid adenosyltransferase	Hearing difficulty	dbSNP, GTEX	rs1558804	12	109,788,598–110,042,348	1.57E−10
*LMX1A*	LIM homeobox transcription factor 1-alpha	Hearing difficulty	Proximity, literature	rs7525101	1	165,087,254–165,112,224	4.58E−10
*EYA4*	Eyes absent homolog 4	Hearing aid^*a*^ Hearing difficulty^*b*^	dbSNP, literature	rs9321402^*a*^ rs9493627^*b*^	6	133,789,728–133,812,872	1.85E−09^*a*^ 3.49E−13^*b*^
*MMP2*	72 kDa type IV collagenase	Hearing difficulty	Proximity, literature	rs78528263	16	55,474,324–55,507,592	2.41E−09
*LMO7*	LIM domain only protein 7	Hearing difficulty	dbSNP, literature	rs920701	13	76,343,051–76,455,056	7.04E−09
*SYNJ2*	Synaptojanin-2	Speech-in-noise^*a*^ Hearing difficulty^*b*^	dbSNP, literature	rs138501510^*a,b*^	6	158,600,947–158,600,947	1.08E−08^*a*^ 1.12E−13^*b*^
*GRM5*	Metabotropic glutamate receptor 5	Hearing difficulty	Proximity, literature	rs12785875	11	88,486,635–88,537,621	1.46E−08
*TYR*	Tyrosinase	Hearing difficulty	Proximity, literature	rs12785875	11	88,486,635–88,537,621	1.46E−08
*NOX4*	NADPH oxidase 4	Hearing difficulty	Proximity, literature	rs12785875	11	88,486,635–88,537,621	1.46E−08

^*a,b*^ denote that associations were found for two traits and notate the corresponding strongest associated lead SNPs and *p* values. Lead SNPs were generated with the clumping algorithm in Plink v1.90b3n [116]. Clumping takes into consideration the linkage disequilibrium between proximal associated SNPs and determines the strongest associated independent SNPs within a genomic region. These are referred to as “lead SNPs”

### Expression of hearing-trait associated proteins in the human cochlea and contrasts with previous results

**Fig. 1:**
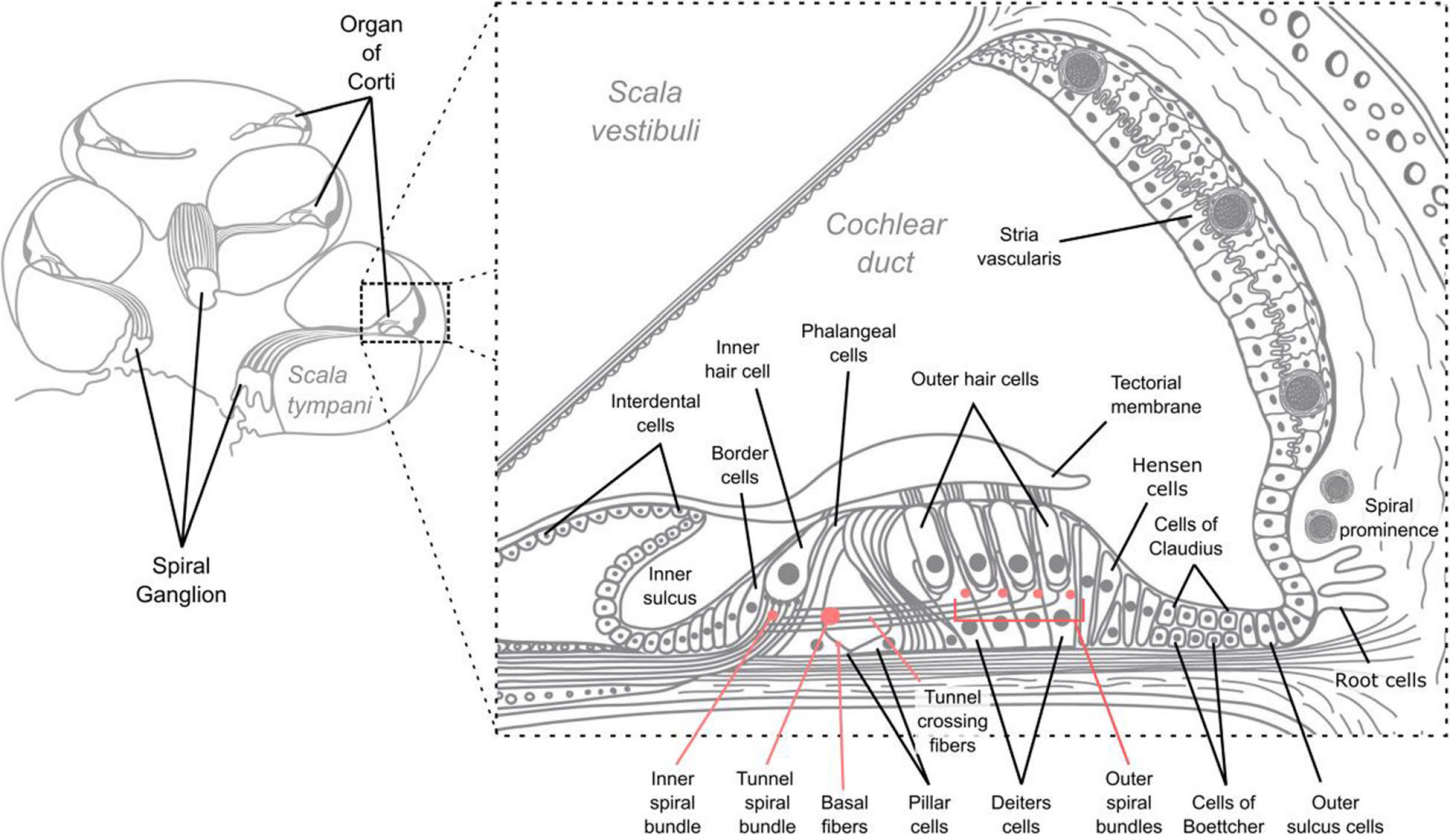
Overview of the human cochlea and organ of Corti. Red is used as a contrast to better visualize the inner and outer spiral bundle, tunnel spiral bundle, and basal fibers. The cells of Claudius were not labeled but are located above the cells of Boettcher. Image adapted with permission from Liu et al. [117]

Immunostaining and imaging of human cochlear sections was informative for all ten selected proteins. The results from immunohistochemical staining are summarized in Table 2. A schematic overview of the human cochlea and organ of Corti is presented in Fig. 1.

**Table 2:** Summary of human cochlear expression of proteins linked to hearing-related traits in the UK Biobank

	Spiral ganglion		Hair cells	*Scala media*-facing epithelial cells											
Protein	Type I neurons	Outer spiral bundles	Inner	Outer	Inner sulcus cells	Inter-dental cells	Border cells	Phalangeal cells	Pillar cells	Deiters cells	Hensen cells	Boettcher cells	Claudius cells	Outer sulcus cells	Stria vascularis
EYA4	+	−	+	+	+	+	+	+	+	+	+	+	+	+	(−)
LMX1A	+	−	−	−	−	−	−	−	+	(+)	+	(−)	(−)	(−)	(−)
PTK2/FAK	+	+	−	−	−	−	−	−	−	−	−	(−)	(−)	(−)	(−)
UBE3B	+	−	−	−	−	−	−	−	−	−	−	−	−	−	(−)
MMP2	+	+	−	−	(−)	(−)	(−)	(−)	(−)	−	−	(−)	(−)	(−)	(−)
SYNJ2	+	+	(−)	−	(−)	(−)	(−)	(−)	−	−	−	(−)	(−)	(−)	(−)
GRM5	+	(−)	(−)	(−)	(−)	(−)	(−)	(−)	(−)	(−)	(−)	(−)	(−)	(−)	(−)
TRIOBP	+	+	+	+	−	−	−	−	−	−	−	(−)	(−)	(−)	(−)
LMO7	(+)	(+)	+	+	(−)	(−)	(−)	−	−	−	(−)	(−)	(−)	(−)	(−)
NOX4	+	−	−	+	+	+	+	+	+	(+)	+	(−)	(−)	+	+

“+” positive protein expression detected by immunohistochemical staining. “−” expression was not detected in this structure. (+) Weak staining in the immunohistochemical imaging suggests the presence of protein in the structure. (−) expression was not detected in this structure (data not presented in this paper)

### LMO-7

**Fig. 2:**
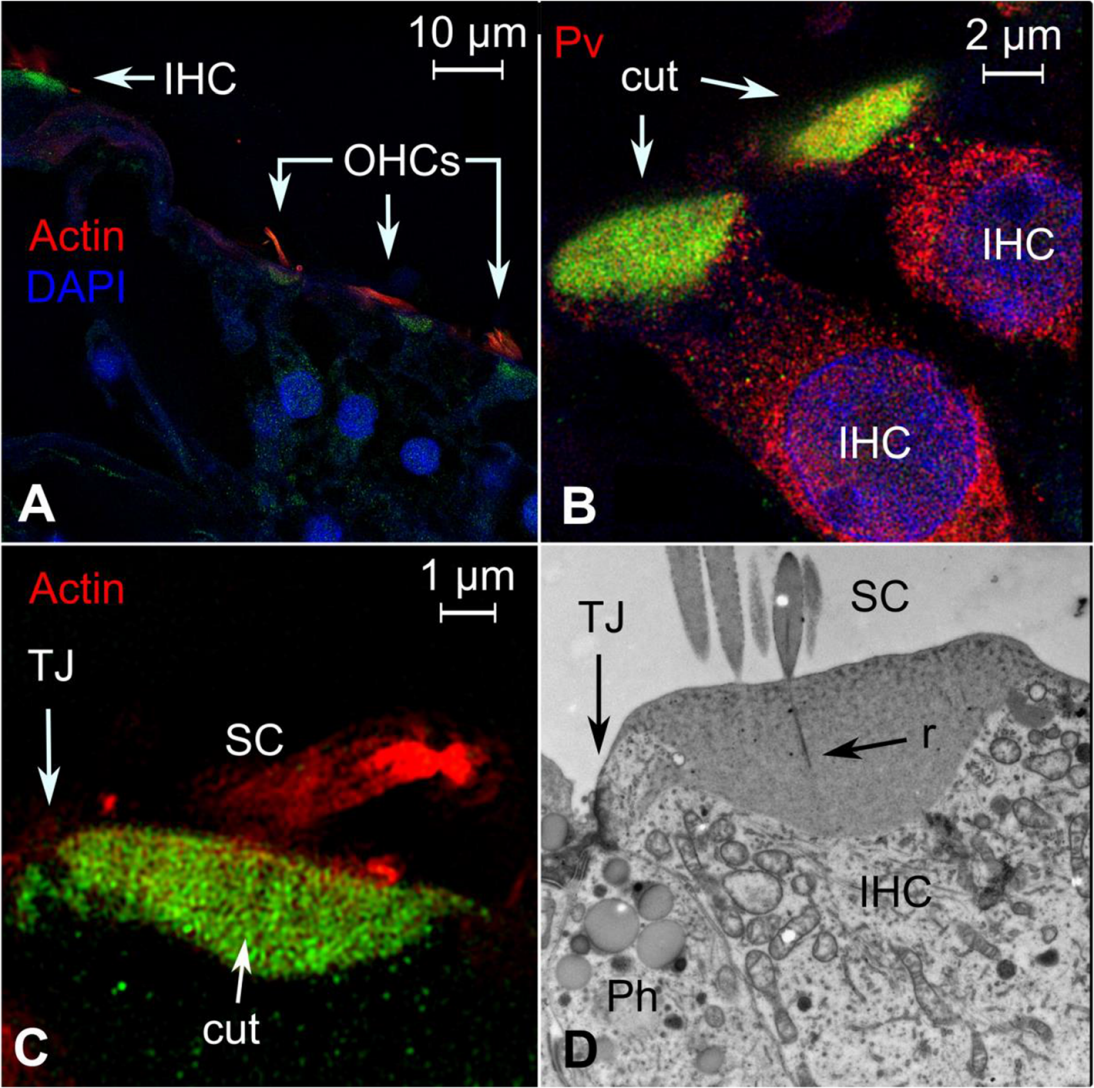
Expression pattern of LMO7 in the human cochlea. **A** Super-resolution structured illumination microscopy (SR-SIM) shows LMO7 (green) is expressed in the cuticular plates of the inner and outer hair cells. **B** Higher magnification of two tangentially cut inner hair cells. Parvalbumin is expressed in the cell bodies and LMO7 in the cuticular plates. **C** Close-up view of the cuticular plate of an inner hair cell. Actin staining was observed in the stereocilia and cuticular plates. LMO7 is only expressed in the cuticular plates and to a lesser degree in the cytoplasm, particularly near the tight junction between the inner hair cell and phalangeal supporting cell. **D** Corresponding transmission electron microscopy (TEM) of a human inner hair cell. The stereocilia contain central rootlets that extend into the cuticular plate. A large number of mitochondria face the apical pole and the cuticular plate. The cuticular plate extends laterally against the junctional area, as shown in **C**. This figure is a modified version of a previously published TEM image [117] and is used here with permission. IHC = inner hair cell, OHCs = outer hair cells, Pv = Parvalbumin, cut. = cuticula, TJ = tight junction, SC = stereocilia, Ph = phalangeal cell, r = stereociliar rootlet

We observe LIM domain only protein-7 (LMO-7) to be expressed in the cuticular plate of the human outer and inner hair cells (Fig. 2A–C). LMO-7 expression also extended laterally to the junction between the inner hair cell and the surrounding supporting cells (Fig. 2C, D). The pattern of expression was consistent with murine expression in the cuticula and cell junctions of both the outer and inner hair cells [80]. *Lmo7*-deficient knockout mice exhibited decreased density of filamentous actin, thinning of the cuticula, and hair cell deformities along with progressive hearing loss leading to profound deafness [80]. In vitro experiments on COS-7 cells showed human LMO7 protein to condense filamentous actin [80]. In the cuticula, actin forms a dense filamentous network, which provides a stable mechanical foundation for stereocilia rootlets [80].

### TRIO and F-actin-binding protein (TRIOBP)

**Fig. 3:**
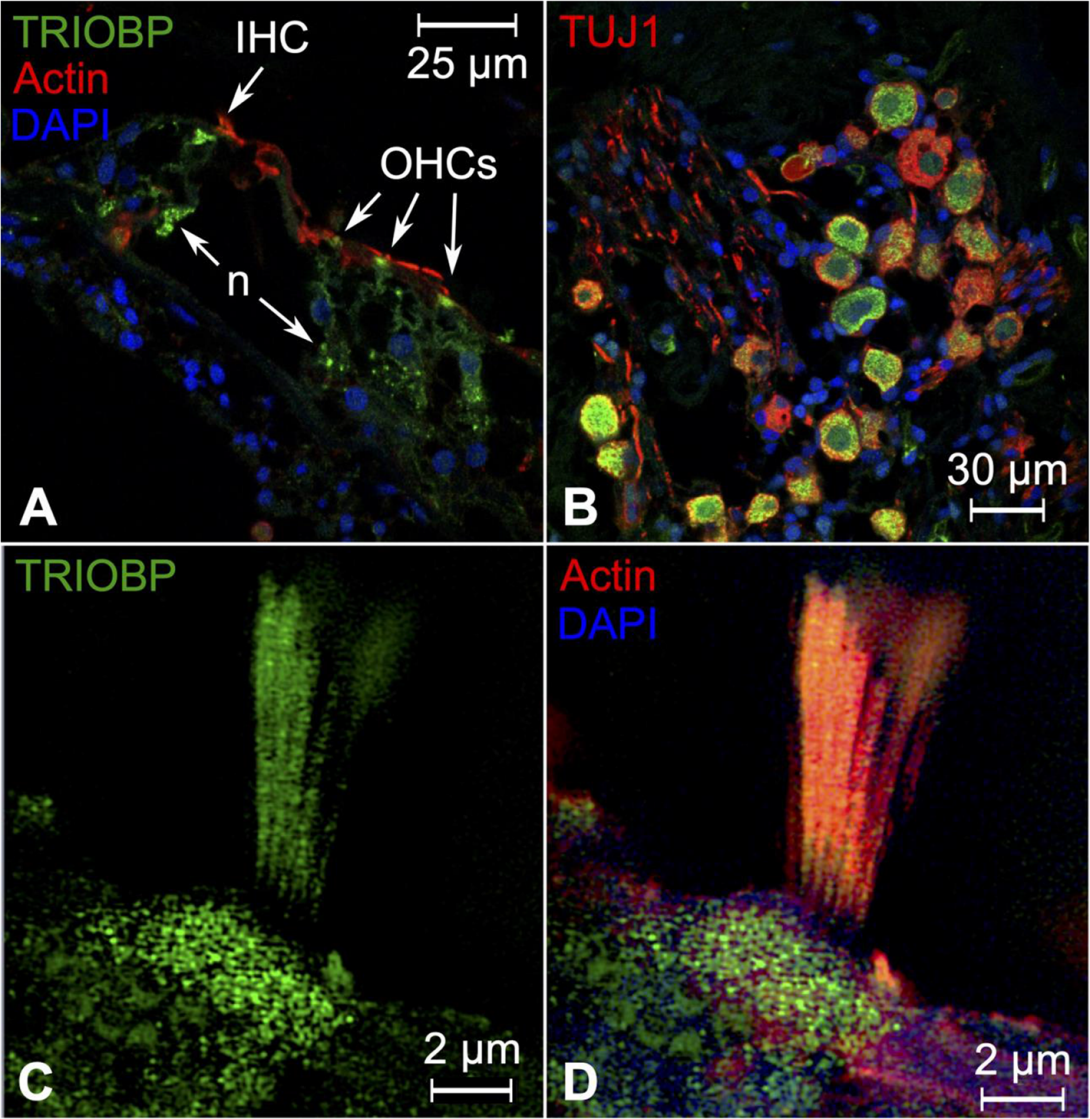
Expression pattern of TRIOBP in the human cochlea. **A** SR-SIM image that illustrates TRIOBP expression in the organ of Corti. Expression is present in inner and outer hair cell bodies, cuticula, stereocilia bundles, and nerve fibers. **B** Confocal microscopy of human spiral ganglion cell bodies expressing TRIOBP. Expression is not seen in the central axons. **C** Higher magnification shows TRIOBP expression in outer hair cell stereocilia and cuticula. **D** Corresponding image shows co-expression of actin and TRIOBP in the stereocilia bundle and cuticular plate. IHC = inner hair cell, OHCs = outer hair cells, n = afferent/efferent neurons, TUJ1 = neuron-specific class III beta-tubulin. Nuclei are stained with DAPI (blue)

TRIOBP expression could be observed in the cuticular plates of the inner and outer hair cells: in the cell bodies and stereocilia, and in the nerve fibers that innervate the inner and outer hair cells, as well as the outer spiral bundle (Fig. 3A, C, D). Expression of TRIOBP was also observed in type I spiral ganglion neuronal cell bodies (Fig. 3B). Cuticula and stereocilia exhibited co-expression of TRIOBP and actin (Fig. 3C, D). The expression pattern in the human cochlea was consistent with murine expression in the inner and outer hair cell stereocilia [42]. In the hair cell, TRIOBP condensates actin into high-density paracrystalline bundles, which extend from the stereocilia into the cuticula [42]. This structure has been termed the rootlet and likely functions as a mechanical support during soundwave-induced deformation and pivoting of the stereocilia. The function of TRIOBP in type I spiral ganglion neurons has not been characterized but is likely to also involve organization of the actin cytoskeleton. The lead SNP at this locus, rs739138, is a missense variant within exon 6 and would thus affect the translated sequences of *TRIOBP* splice variant isoforms 5 and 4. Non-syndromic autosomal recessive deafness 28 (DFNB28) has also been mapped to nonsense and frameshift mutations that occur mainly in exon 6 of *TRIOBP* [33, 34].

Both humans and mouse exhibit three protein isoforms of TRIOBP: TRIOBP-5, which is encoded by a transcript that includes exons 1–3, 5–9, and 12–24, TRIOBP-4, which is encoded by a transcript that ends after exon 6, and TRIOBP-1, which is encoded by a transcript that only includes exons 11–24 [42]. Of these three isoforms, TRIOBP-5 and TRIOBP-4 have both been observed in the stereocilia rootlet [42]. The longest of these two isoforms, TRIOBP-5, has been demonstrated to be the most critical for proper development of the stereocilia rootlets and stereocilia function [118]. The expression patterns of these two isoforms also differ: TRIOBP-5 expression was observed only in the bottom half of the rootlet within the cuticula while TRIOBP-4 expression extended the full length of the rootlet [118]. It should be noted that the antibody used in this study (Novus Biologicals: NBP1-90590) targets an amino acid sequence that spans the translated sequence of exons 20–23 of *TRIOBP*. Consequently, the results presented in this study reflect the expression patterns of TRIOBP isoforms 5 and 1, which both include these exons.

### Eyes absent homolog 4 (EYA4)

**Fig. 4:**
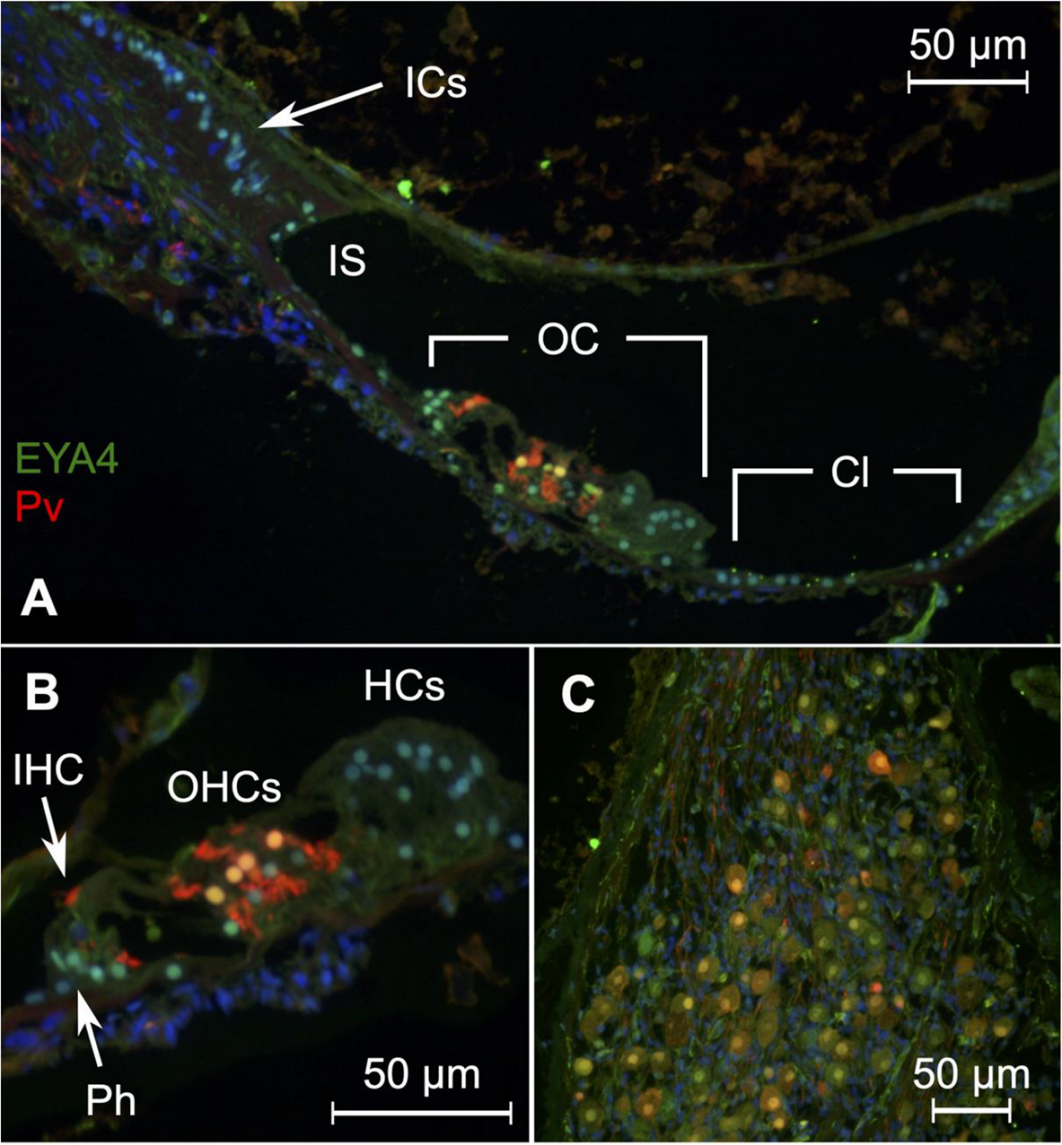
Expression pattern of EYA4 in the human cochlea. **A** EYA4 expression is observed in membranous labyrinth supporting cell nuclei: within the organ of Corti, the inner and outer sulcus, cells of Claudius, and spiral limbus interdental cells. Parvalbumin stains the inner and outer hair cells red. Nuclei were stained with DAPI. **B** Close-up of the organ of Corti reveals EYA4 expression in the outer hair cells as well as supporting cells: phalangeal cells, pillar cells, Deiters cells, Hensen cells, and Boettcher cells. Cells beneath the basilar membrane lack EYA4 expression. **C** EYA4 is expressed in the spiral ganglion cells. ICs = inderdental cells, IS = inner sulcus, OC = organ of Corti, Cl = cells of Claudius, IHC = inner hair cell, OHCs = outer hair cells, HCs = Hensen cells, Ph = phalangeal cells

EYA4 was observed to be expressed in supporting cells of the membranous labyrinth and organ of Corti: in the interdental cells, inner sulcus cells, phalangeal cells, pillar cells, Deiters cells, Hensen cells, Boetcher cells, and cells of Claudius; as well as in the nuclei of the outer and inner hair cells (Fig. 4A, B). Other cell nuclei such as in the tympanic covering layer and spiral ligament were negative. Expression was also observed in the nuclei of the spiral ganglion cells (Fig. 4C). Expression in the inner and outer hair cells, as well as supporting cells and spiral ganglion, is consistent with the expression in the cochlea of the common marmoset, *Callithrix jacchus* [32]. We are also able to report EYA4 expression in the wall of the inner spiral sulcus. Human EYA4 expression was particularly strong in cell nuclei, which is consistent with its role as a transcriptional regulator with histone phosphatase activity [119]. *Eya4* expression was determined in the cochlea of the common marmoset due to the phenotypic discrepancies between *Eya4*-deficient mice and humans with EYA4 linked non-syndromic autosomal dominant deafness 10 (DFNA10). *Eya4*-deficient mice exhibit Eustachian tube dysmorphology and otitis media with effusion [120] which is not present in DFNA10 humans. *Eya4* RNA expression studies in the developing rat cochlea indicate that this protein is important for normal development of the cochlea [13]. Our findings, as well as those in primates [32], indicate a role also in maintenance of differentiated cellular states in the adult cochlea.

### Co-staining of synaptojanin 2 (ch) and matrix metalloproteinase 2 (MMP2)

**Fig. 5:**
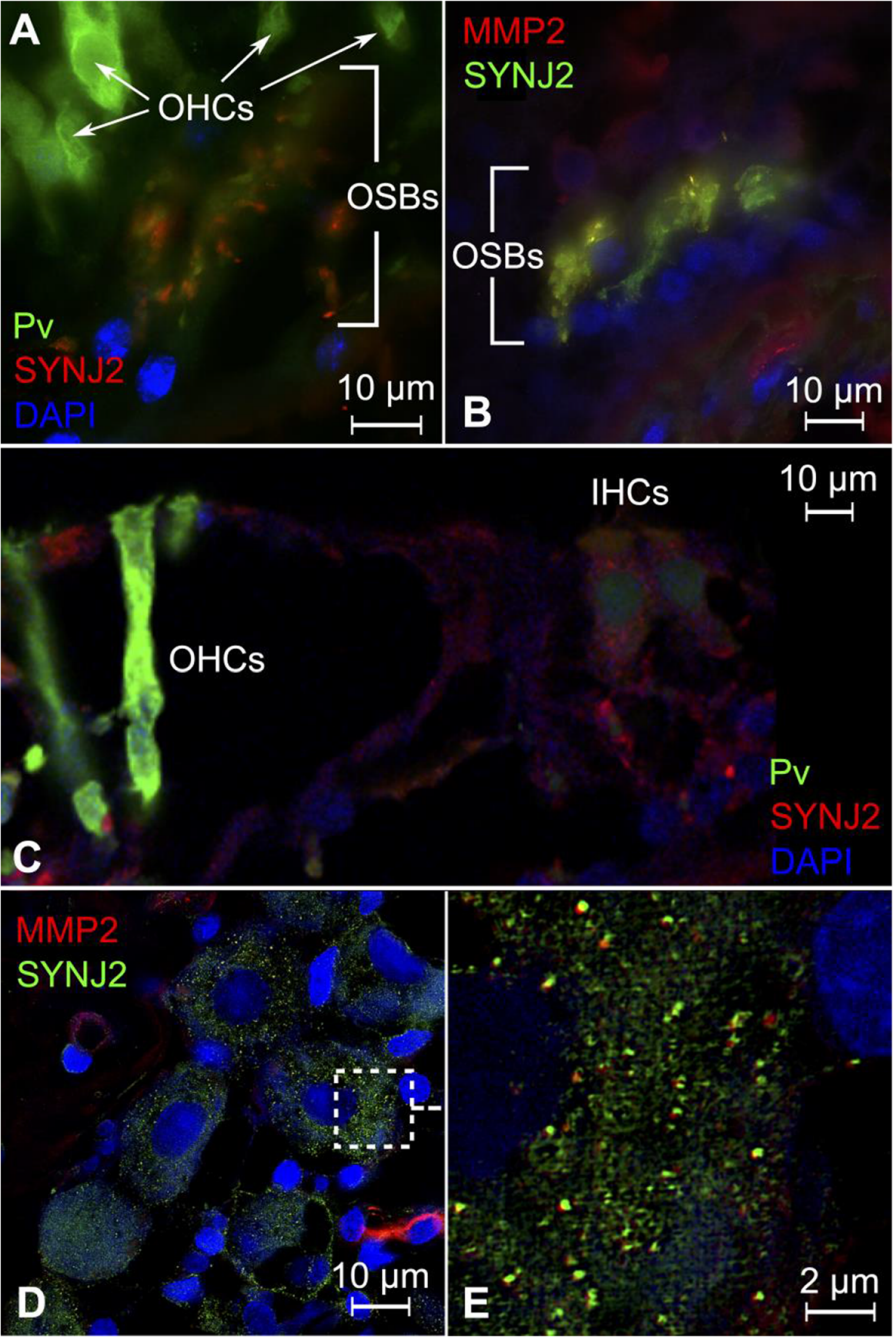
Expression pattern of SYNJ2 and MMP2 in the human cochlea. **A** SR-SIM reveals expression of SYNJ2 in the outer spiral bundles, observed here beneath the outer hair cells, which express parvalbumin. **B** Co-staining of MMP2 and SYNJ2 reveal co-expression within in the outer spiral bundles. **C** Confocal microscopy of outer and inner hair cells showing lack of SYNJ2 expression. Inner hair cells are only weakly stained for SYNJ2, which is believed to be unspecific. **D** Spiral ganglion neurons exhibit MMP2 and SYNJ2 expression. **E** Higher magnification of the area outlined in **C**. SYNJ2 and MMP2 can be observed to colocalize in ~ 100 nm molecular aggregates. Pv = parvalbumin, OHCs = outer hair cells, IHCs = inner hair cells, OSBs = outer spiral bundles

SYNJ2 was observed to be expressed in the outer spiral bundles (Fig. 5A). Co-staining with MMP2 reveals co-expression with SYNJ2 in the outer spiral bundles (Fig. 5B) and the cytoplasm of spiral ganglion neuronal cells (Fig. 5D, E). Higher magnification suggests that SYNJ2 and MMP2 colocalize in ~ 100-nm molecular aggregates within the spiral ganglion neuronal cytoplasm (Fig. 5E). These findings contrast previous findings in mice, where *Synj2* RNA was found to be expressed in the outer and inner hair cells [44] as well as the spiral ganglion [114] (Fig. 5C). *SYNJ2* encodes an inositol 5-phosphatase enzyme, which de-phosphorylates phosphatidylinositol (3,4,5)-triphosphate (PIP_3_) and phosphatidylinositol (4,5)-biphosphate, and plays a key role in recovery of secretory vesicles in presynaptic neurons via endocytosis [121]. The mouse homolog *Synj2* was linked to progressive hearing loss in the Mozart mouse strain, which was generated in a N-ethyl-N-nitrosourea (ENU) mutagenesis program at the Australian Phenomics Facility in Canberra [44]. This strain carries a mutation within the phosphatase catalytic domain of *Synj2* and are born with normal hearing that deteriorates by 8 weeks, leading to complete loss of hearing at 12 weeks. Mozart mice also exhibit stereociliar fusion, loss of hair cell bundles, and subsequent hair cell degeneration [44]. A second study on mice with a different *Synj2* mutation in the phosphatase catalytic domain was able to confirm the observation of progressive hearing loss in *Synj2-*deficient mice [114].

Expression of MMP2 in the cytosol of type I spiral ganglion neuron was consistent with imaging in the rat spiral ganglion [73, 112]. We are also able to report expression of MMP2 in the outer spiral bundle. Cytosolic co-staining of MMP2 and SYNJ2 in spiral ganglion neuronal cells may represent MMP2-containing secretory vesicles. MMP2 is a matrix metalloproteinase that degrades type IV collagen, which may play a role in the elongation of neuronal outgrowths during development and extracellular matrix remodeling after tissue damage. Several experiments in guinea pig and rat models for cochlear damage have demonstrated that that *Mmp2* RNA expression increases after noise exposure [71], ototoxic aminoglycoside antibiotics [73, 112], and cochlear implantation [72]. Aminoglycosides also increased the permeability of the osseous spiral lamina in rat cochlea, which led to increased diffusion across *canaliculi perforantes* from the *scala tympani* and *scala vestibuli* to spiral ganglion neurons embedded in Rosenthal’s canal [73]. This effect was attenuated by concomitant administration of the metalloproteinase inhibitor oxytetracycline [73]. Metalloproteinase inhibitors ilomastat and oxytetracycline were also protective against middle ear inflammation-induced hearing loss in guinea pigs that underwent intratympanic instillation of bacterial endotoxins [74].

### Ubiquitin-protein ligase E3B (UBE3B)

**Fig. 6:**
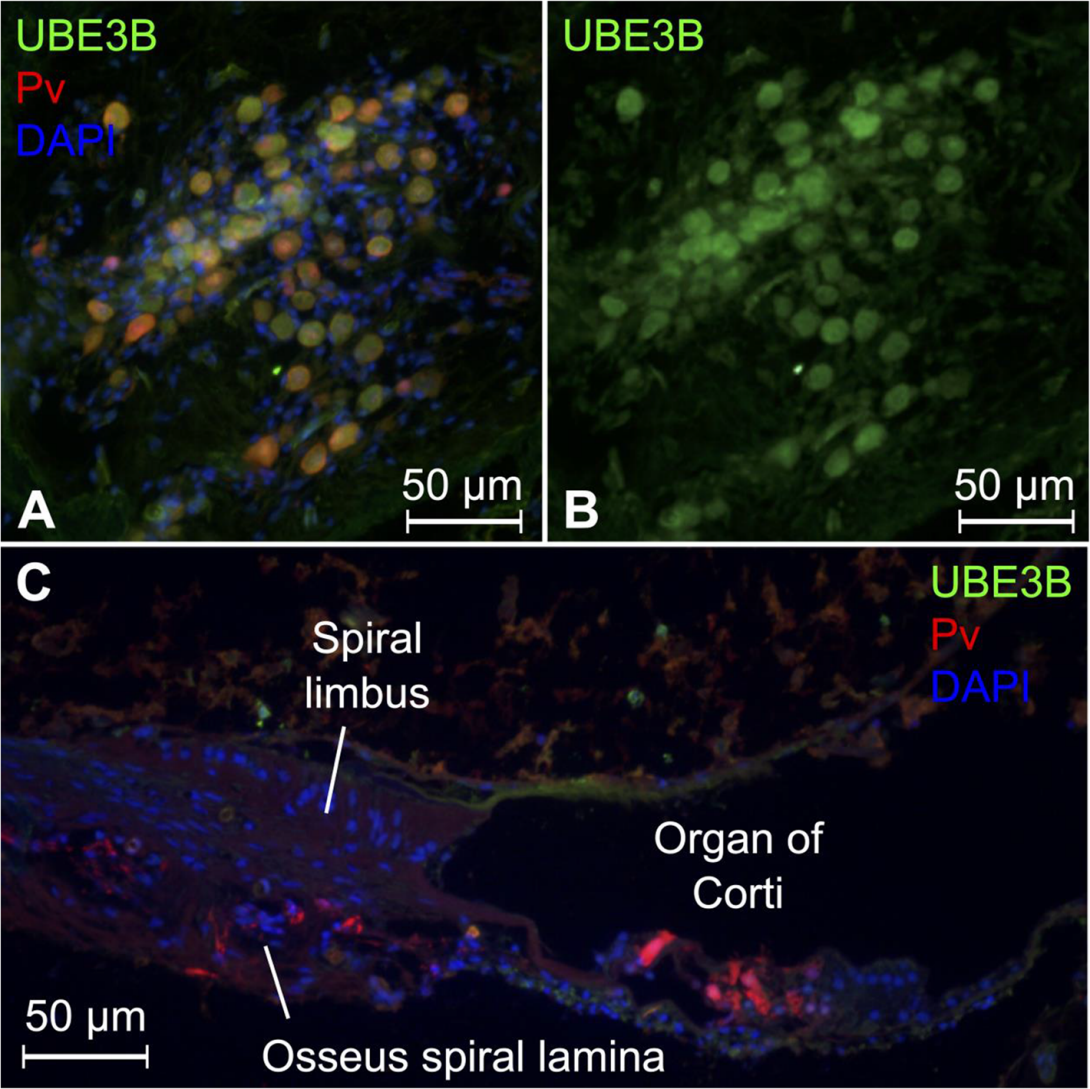
Expression pattern of UBE3B in the human cochlea. **A** UBE3B is expressed together with parvalbumin in human spiral ganglion cell bodies in the basal modiolus. **B** Same image as **A** but showing UBE3B staining without the neuronal marker (Pv) and DAPI. Pv = parvalbumin. **C** Low power image of a radial section of the cochlear duct. Hair cells and neural elements stain positive for parvalbumin. UBE3B was not detected

UBE3B expression was observed in both the nuclei and cytoplasm of myelinated neurons of the spiral ganglion (Fig. 6A, B). Expression was not observed in the cochlear duct (Fig. 6C). Previous studies have reported induced expression of *Ube3b* RNA in the chick basilar papilla, the auditory sensory organ of birds, lizard, and amphibians, following noise exposure [57], as well as in a wide expression of *Ube3b* RNA across an array of twenty mouse tissues [122]. Histological imaging of UBE3B protein in the cochlea or auditory organ for any species has previously not been published. Loss of function mutations within *UBE3B* have been linked to Kaufman oculo-cerebro-facial syndrome, a rare recessive congenital disorder that is mainly characterized by microcephaly, ocular anomalies, intellectual disability, and distinctive facial features, where hearing loss also has been reported [55, 56]. *UBE3B* encodes a calmodulin-regulated E3 ubiquitin ligase with a putative role in maintaining mitochondrial integrity during oxidative stress via ubiquitin-tagging of damaged proteins for degradation via the 26S proteasome [123].

### Focal adhesion kinase 1 (FAK)

**Fig. 7:**
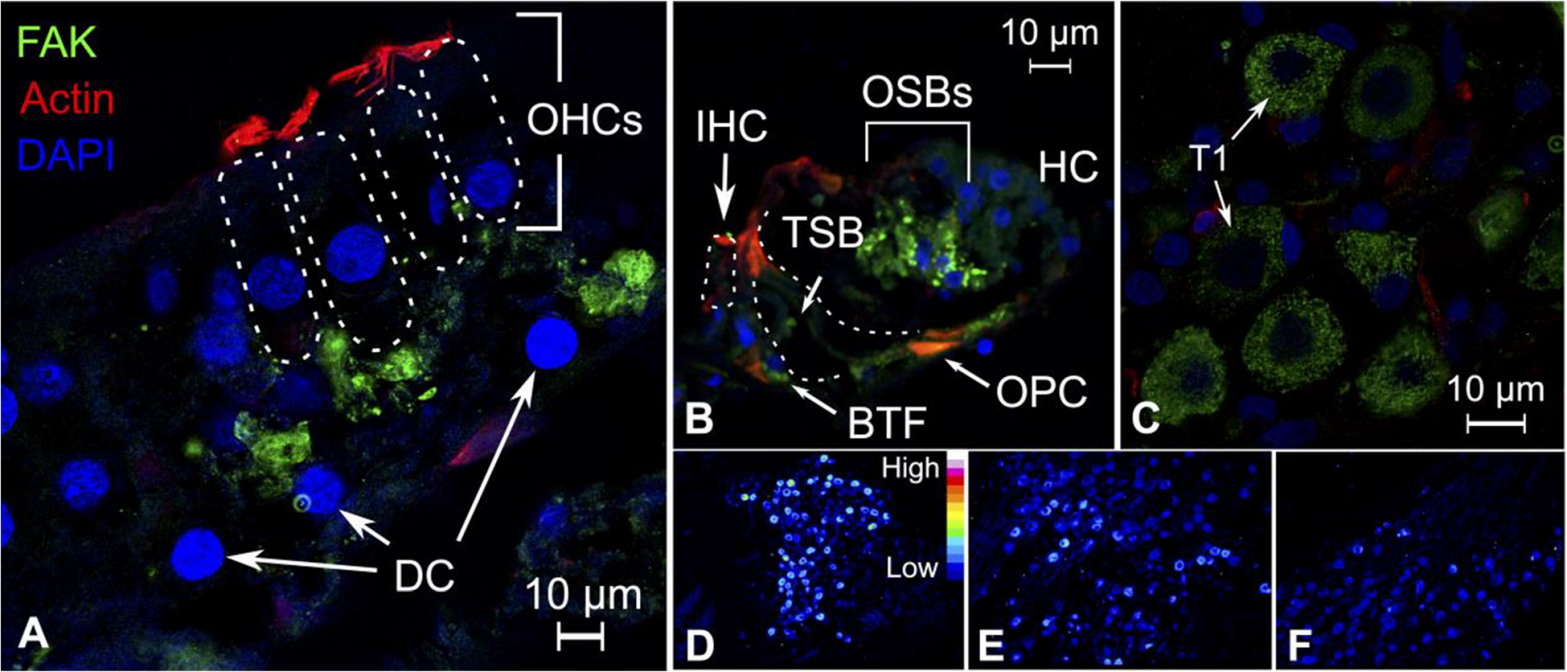
Expression pattern of PTK2/FAK in the human cochlea. **A** FAK expression was observed in both afferent and efferent neurons that innervate the hair cells. Actin was also present in the hair cell stereocilia and cuticular plates. The outer hair cells are highlighted with broken lines. **B** Low-power confocal image shows FAK staining of basal tunnel fibers and the tunnel spiral bundle. Actin-positive areas of the head and foot regions of the pillar cells are shown. Pillar cells contain invaginated afferent neurons (basal tunnel fibers) that innervate the outer hair cells. The inner hair cell and pillar cells are highlighted with broken lines. **C** Expression of FAK was present in the cytoplasm of spiral ganglion cells. **D–F** False color imaging reveals a gradient expression of FAK from the cochlear base to the apex. High expression intensity was observed in the cochlear base (**D**), which gradually decreases in neurons that supply the second (**E**) and third cochlear turns (**F**). OHCs = outer hair cells, T1 = type I spiral ganglion cells, or alternatively, large spiral ganglion cells. DC = Deiters cells, OPC = outer pillar cells, BTF = basal tunnel fibers, TSB = tunnel spiral bundles, HC = Hensen cells, OSBs = outer spiral bundles, IHC = inner hair cell

FAK-stained positive in the outer spiral bundles beneath the outer hair cell layer (Fig. 7A, B). Basal tunnel fibers and tunnel spiral bundles were also positive for FAK. FAK expression was also observed in spiral ganglion neurons but not in surrounding glial and mesenchymal cells (Fig. 7C). False color imaging also revealed FAK to exhibit a gradual decrease in expression from the basal turn of the cochlea to the apex (Fig. 7D-F). A previous study on the cochlea of the long-haired chinchilla (*Chinchilla lanigera*) reported FAK protein expression in the supporting cells of the organ of Corti [111]. Expression was also induced in damaged outer hair cells following noise exposure [111]. It is possible that our extraction of human cochleae has induced compensatory mechanisms within the cochlea, which may include increased FAK expression. The surgical procedure to remove petroclival meningiomas via the transpetrosal approach includes careful drilling over an extended time period, which exposes the cochlea to loud noise and vibrations. This may explain our observations of a gradual increase of FAK expression in type I human spiral ganglion neurons from the cochlear apex to the basal turn, as the basal regions of the cochlea is more sensitive to noise.

### Metabotropic glutamate receptor 5 (GRM5)

**Fig. 8:**
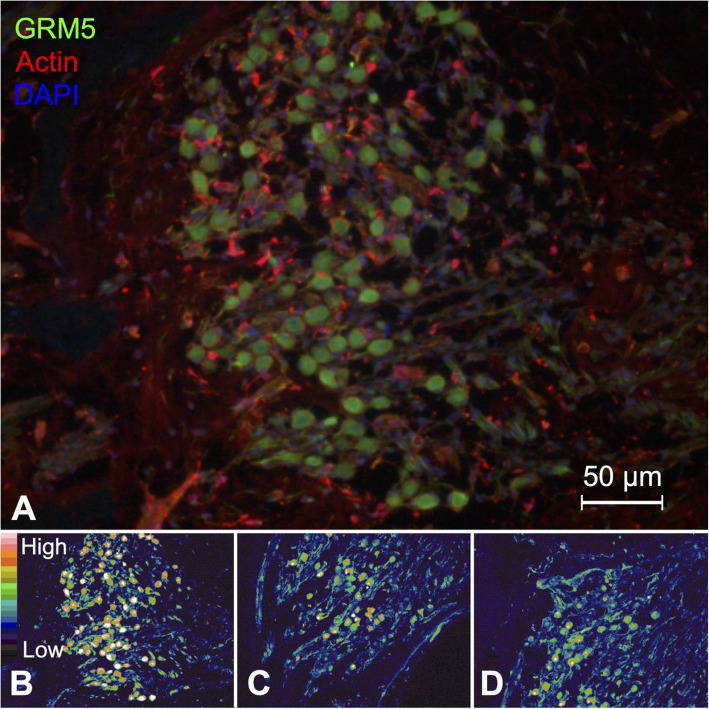
Expression pattern of GRM5 in the human spiral ganglion. **A** GRM5 was observed in spiral ganglion cells. **B–D** A gradient expression of GRM5 was observed: from high expression in the basal turn (**B**) of the cochlea, which diminished gradually towards the midturn (**C**) and cochlear apex (**D**)

GRM5 was also expressed in the spiral ganglion cells (Fig. 8A). Similar to FAK, false color imaging revealed a gradient increase in GRM5 expression from the basal turn of the cochlea to the apex (Fig. 8B-D). *GRM5* encodes the metabotropic glutamate receptor 5, which is a postsynaptic G protein-coupled receptor that activates downstream signaling via phospholipase C after glutamate-binding to the extracellular portion of the receptor. There is no previous data on GRM5 expression in the cochlea, in humans or any other species. However, metabotropic glutamate receptor 5 signaling has been linked to central auditory processing pathways from the medial geniculate nucleus in the brainstem, to the lateral amygdala in rats [89, 90, 124]. Grm5 has also been linked to enhancement of glutamate signaling in the medial nucleus of the trapezoid body in mice (MNTB) [125]. This region transmits auditory information from the cochlear nucleus to the superior olivary complex and is involved in sound source localization [126].

### LIM homeobox transcription factor 1-alpha (LMX1A)

**Fig. 9:**
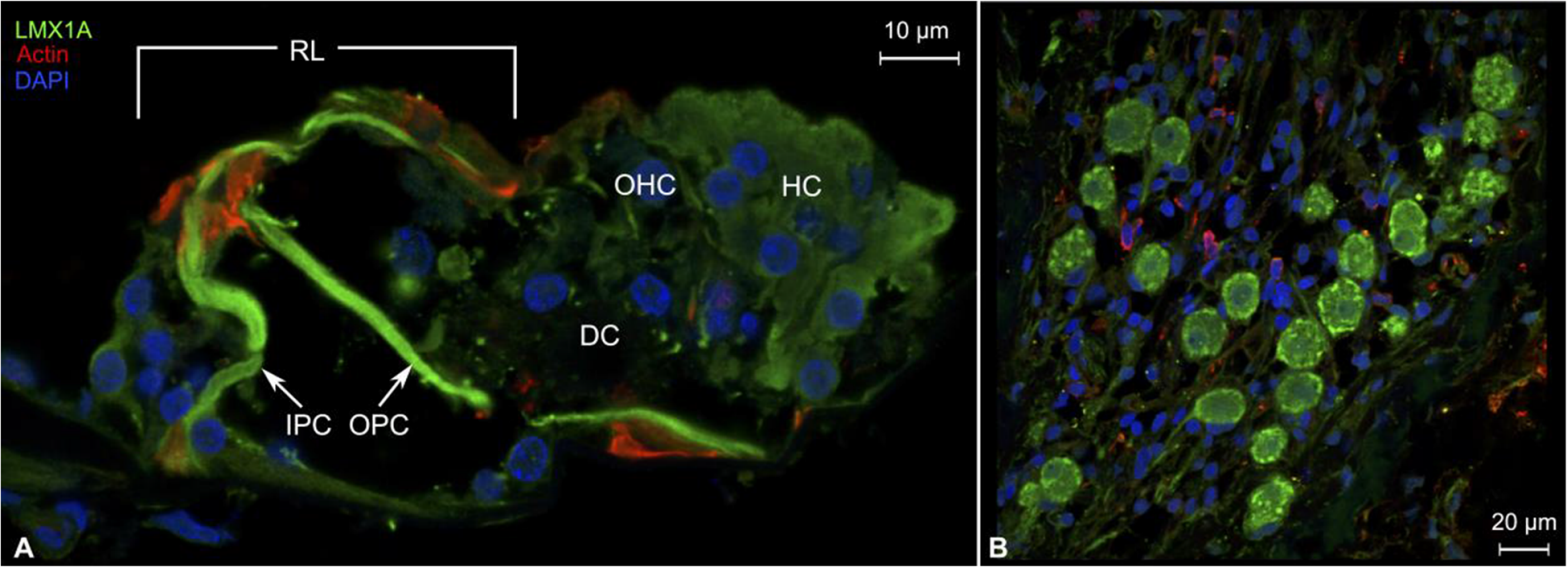
Expression pattern of LMX1A in the human cochlea. **A** Expression of LMX1A and actin in the organ of Corti. LMX1A was expressed in both the inner and outer pillar cells, as well as the reticular lamina and Hensen cells. Weak staining is visible in Deiters cells, which suggests the presence of LMX1A also in these cells. Actin expression was observed in the pillar cell feet and heads. Actin was also visible in the stereocilia of the hair cells. **B** LMX1A was also expressed in the spiral ganglion neurons, with increased expression in the peripheral regions of the cell body cytoplasm. IPC = inner pillar cell, OPC = outer pillar cell, RL = reticular lamina, OHC = outer hair cell, DC = Deiters cell, HC = Hensen cells

LMX1A expression was observed in the inner and outer pillar cells and the reticular lamina in the organ of Corti (Fig. 9A). Type I spiral ganglion cell soma cytoplasm also stained positive for LMX1A and exhibited focal accumulations of increased expression in the peripheral regions of the cell bodies (Fig. 9B). Expression in human type I spiral ganglion neurons is consistent with previous results in mice that used in situ hybridization and single-cell RNA analysis to demonstrate *Lmx1a* expression in a discrete type I spiral ganglion neuron subpopulation in the developing mouse cochlea [127]. These subtypes have been hypothesized to correspond to different excitation potentials thresholds [127] and enable further encoding of auditory information at the level of the spiral ganglion [128]. *LMX1A* encodes a transcription factor, LIM homeobox transcription factor 1-alpha, which is involved in neuronal differentiation as well as maintenance of neuronal homeostasis in the adult brain [129]. In mice, LIM homeobox transcription factor 1-alpha has been suggested to delineate non-sensory from sensory epithelia during cochlear development as *Lmx1a*^*-/-*^ mutant mice exhibit severe developmental defects of the cochlea along with disruptions of cochlear cellular organization [68, 130–133]. Our results suggest a role for LMX1A also in the adult human cochlea: in non-sensory supporting cells of the organ of Corti, as well as type I spiral ganglion neurons.

### NADPH oxidase 4 (NOX4)

**Fig. 10:**
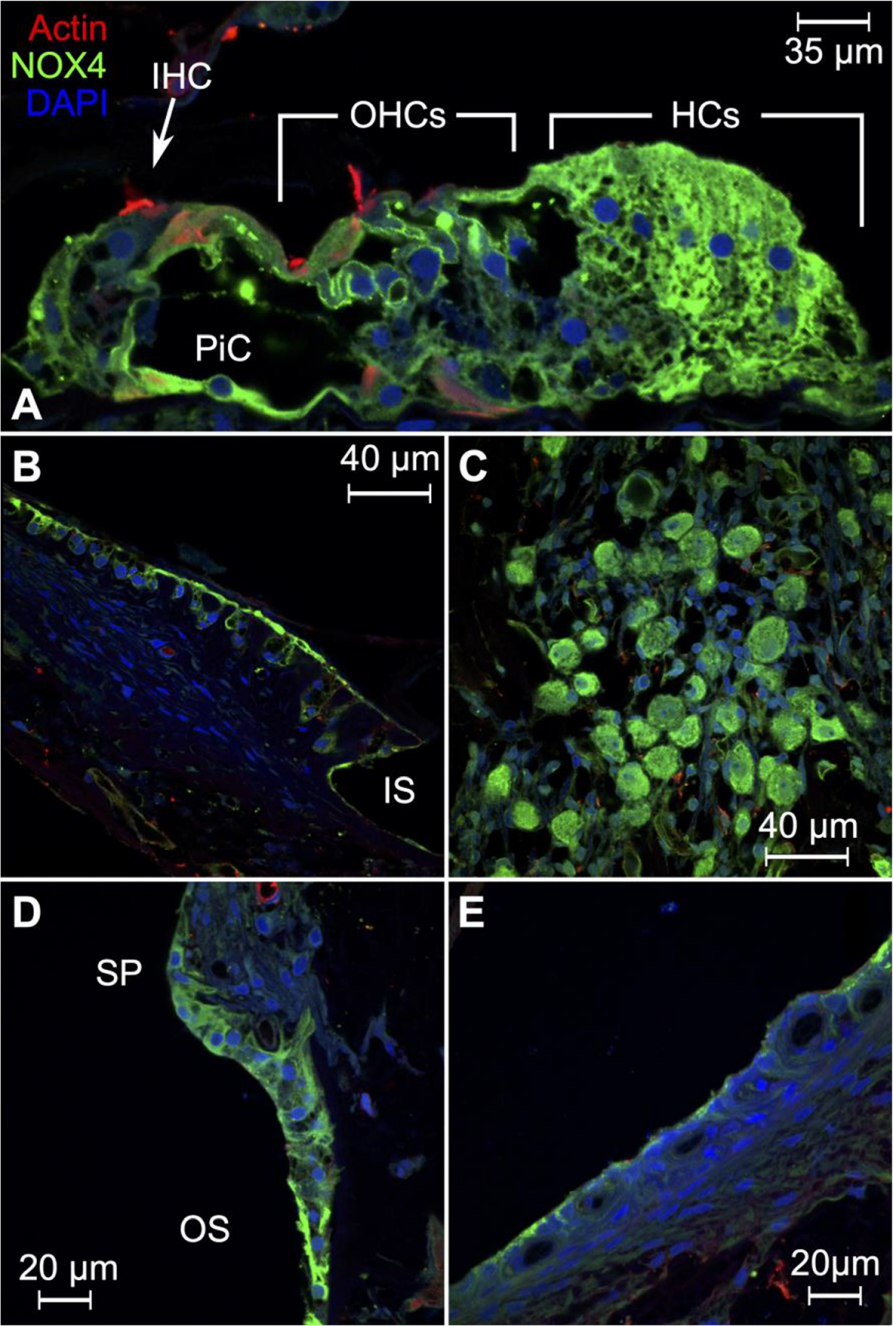
Expression pattern of NOX4 in the human cochlea. **A** Confocal microscopy reveals strong NOX4 expression in supporting cells of the organ of Corti. Particularly strong expression was observed in Hensen cells, pillar cells, and the cell membrane of the outer hair cells. In contrast, NOX4 expression was negative in the inner hair cell. **B** NOX4 is expressed in the interdental cells of the spiral limbus, as well as the inner sulcus cells. **C** Spiral ganglion cells also express NOX4. **D** Expression of NOX4 in the outer sulcus cells and inferior region of the spiral prominence. **E** NOX4 expression was also observed in the apical cell membrane of the marginal cells of the stria vascularis. IHC = inner hair cell, OHCs = outer hair cells, HCs = Hensen cells, PiC = pillar cell, IS = inner sulcus, SP = spiral prominence, OS = outer sulcus

NOX4 was observed in the supporting cell of the organ of Corti with particularly strong expression in Hensen cells, pillar cells, and the cell membrane of the outer hair cells. Interestingly, NOX4 staining in the inner hair cells was negative, while expression was observed in the outer hair cell membrane and cochlear supporting cells (Fig. 10A). NOX4 was observed in the interdental and inner sulcus cells (Fig. 10B), spiral ganglion cells (Fig. 10C), spiral prominence and outer sulcus cells (Fig. 10D). NOX4 expression was also observed selectively in the apical membrane of the marginal cells of the stria vascularis (Fig. 10E). Cochlear NOX4 expression has not previously been described in other species. *NOX4* encodes the NADPH oxidase 4 enzyme, which catalyzes the reduction of di-oxygen (O^2^) to super-oxide (2O^•−^), a precursor of reactive oxygen species. Reactive oxygen species are canonically known to damage protein structures within the cell. However, at low concentrations, reactive oxygen species also act as intracellular signaling molecules [134]. In vitro experiments have revealed a role for NOX4 in regulation of cytoskeletal actin structures. In human cell lines, downregulation of *NOX4* by RNA interference led to a reduction in filamentous actin stress fibers and reduced cellular migration [135].

### Contrasts with previous GWAS

The results from our study are consistent with the recent GWAS of hearing difficulty and hearing aid use on UK Biobank participants [7]. However, some differences in the results are present, which are most likely due to methodological differences in the definition of phenotypes and the statistical analyses: First, Wells et al. used a more stringent inclusion criteria to exclude low frequency SNPs below 1%, while our analyses included low-frequency SNPs above 0.01%. Secondly, BOLT-LMM [136] was used to model SNP effects, which contrasts our use of PLINK. And third, Wells et al. used a more stringent criteria for hearing difficulty by filtering cases and controls for whether they self-reported hearing difficulties with background noise (data field 2257: “Do you find it difficult to follow a conversation if there is background noise (such as TV, radio children playing)?”), and also excluded participants under the age of 50 for the hearing difficulty phenotype to better match the age distribution for the hearing aid use phenotype. Data for individuals that were assessed more than once was also recoded to use the most recently recorded data. These methodologcal differences led to a lower number of cases and controls in the GWAS for hearing difficulty compared to the analyses presented here (87,056 cases and 163,333 controls in the analysis by Wells et al*.* vs. 91,080 cases and 257,037 controls in the current analysis), as well as a higher number of cases and controls in the GWAS for hearing aid (13,178 cases and 240,740 controls vs. 11,081 cases and 208,083 controls in our analyses). Consequently, twenty hearing difficulty associated and 22 hearing aid use-associated loci that are reported in our study were not observed to be significantly associated in the previous GWAS [7]. Conversely, 18 hearing difficulty associated and four hearing aid-associated loci reported in the recent GWAS were not found to be associated in our analyses. Due to the differences in minor allele frequency cut-offs, we are able to report several associations for low-frequency SNPs, which were excluded in the recent GWAS by Wells et al. Differences between SNPs that were included in both analyses were mainly found for more weakly associated SNPs (Additional file 1: Fig. 3), which can be expected to be less robust to any remaining confounding effects.

Among the hearing difficulty associated loci that were not reported by Wells et al. were several highly biologically relevant genes that are unlikely to represent spurious associations, such as *SYNJ2*, *SLC26A5* (prestin, DFNB61), *LMO7*, *SPTBN1 (*DFNA58), and *FSCN2*. Conversely, Wells et al. reported an association of a missense variant within *CDH23*, which encodes a tip-link protein, cadherin 23. This protein forms a link between the tips of the stereocilia, usually from the tallest to the shorter, and mediates mechanotransduction during stereociliar deflection by opening spring-gated ion channels on the lower stereocilia [137]. Mutations in *CDH23* have been found to be causal for the recessive autosomal deafness locus DFNB12 [138].

## Discussion

GWAS of self-reported hearing loss-related traits in the UK Biobank revealed that a substantial amount of the associated loci, approximately 40%, could be linked to genes that were determined to be biologically relevant for human hearing. Furthermore, associations overlapped with human congenital non-syndromic deafness loci and associations were found that were proximal to genes with determined functional roles in human auditory mechanotransduction, such as *SLC26A5*, which encodes the motor protein of the outer hair cells, prestin. A large number of associated loci also contained genes that have previously been linked to hearing-related traits and cochlear structures in vitro and in vivo in other species. Imaging analyses of GWAS-associated genes in human tissue samples are uniquely informative due to the discrepancies in cochlear morphology and protein expression that occur between species. In addition, we were also able to confirm the cochlear expression patterns of several hearing-associated proteins for the first time in human tissue and generate precise mapping of protein expression even at the subcellular level.

Taken together, our findings strongly implicate spiral ganglion function to be critical for hearing loss. Nine of the proteins that were selected for staining were found to be expressed in human type I spiral ganglion neurons: UBE3B, MMP2, PTK2/FAK, GRM5, SYNJ2, LMX1A, EYA4, NOX4, and TRIOBP. Additional genes with neuronal function were also identified from GWAS: *DYRK2* (Dual specificity tyrosine-phosphorylation-regulated kinase 2), *RAB27B* (Ras-related protein Rab-27B), *SEPT11* (Septin-11), *NTRK3* (NT-3 growth factor receptor), *ASTN2* (Astrotactin-2), *BAIAP3* (BAI1-associated protein 3), and *MAST4* (microtubule-associated serine/threonine-protein kinase 4). Similar to the hair cells, spiral ganglion neurons are sensitive to environmental factors such as noise, ototoxic compounds, and inflammation [139]. Damage to the spiral ganglion cells can also occur without apparent damage to the hair cells. In mice, a reversible noise-induced shift in the auditory threshold led to acute loss of synaptic terminals at the inner and outer hair cells [140], which is likely to be caused by glutamatergic excitotoxicity that is induced by hair cell hyperstimulation. Otoacoustic emissions and histological imaging revealed structurally intact hair cells following exposure [140]. However, a delayed degeneration of spiral ganglion neuronal cells along with progressive hearing loss was observed in exposed mice over the following years [140]. The analogous situation in humans would constitute noise exposure-induced threshold shifts during adolescence, for instance after a loud concert, without apparent hearing loss or hair cell damage after recovery, followed by progressive hearing loss that debuts in middle age. Further animal studies have suggested neurotrophin-3 to protect against this type of noise-induced synaptic damage [141], and to facilitate synaptic recovery following noise exposure-induced hearing loss [142, 143]. In light of these observations, it is interesting to note that we observed variants linked to neurotrophic signaling pathways to be associated with hearing aid use: *NTRK3*, which encodes the receptor for the neurotrophic factor neurotrophin-3 (NT-3) and has previously been found to be expressed in the spiral ganglion of rat [144–147] and chicken [148], as well as *RAB27B* (Ras-related protein Rab-27B), which mediates anterograde axonal transport of the BDNF/NT-3 growth factor receptor, TrkB [149]. Associations were also observed that could be linked to genes involved in biological processes related to neuronal plasticity, such as extracellular remodeling (*MMP2*), neuronal migration (*ASTN2* and *FAK*), and cytoskeleton organization and neurite outgrowth (*DYRK2*, *NOX4*, and *SEPT11*).

Our findings also suggest that structural resilience of the human hair cells, and perhaps more critically the sterocilia, is an important factor for the risk of human hearing loss that is affected by common genetic variation. Imaging of GWAS-associated genes in human cochlear tissue showed the actin-associated proteins LMO-7, TRIOBP, and NOX4 to all be expressed in the human hair cells, the hair cell cuticulae, and stereocilia. In addition, five additional structural regulators that are expressed in the hair cells, stereocilia, and cuticula in other species were also associated with hearing-related traits in our analyses: *SPTBN1* (beta-II spectrin) [78], *CDH13* (cadherin-13) [95], *BAIAP2L2* (brain-specific angiogenesis inhibitor 1-associated protein 2-like protein 2) [108], *FSCN2* (fascin-2) [150], and *TPRN* (taperin) [97]. Age-related hearing loss has been hypothesized to involve the accumulated damage to the stereocilia and hair cells due to damaging environmental exposures in an individual’s lifetime [151]. Unlike other non-mammalian species, e.g., reptiles and birds, humans are not able to regenerate lost hair cells, e.g., through differentiation of supporting cells to form new hair cells [152]. Survival of the hair cells is thus critical for maintaining hearing across the lifespan. Our results indicate that differences in resilience to structural damage, e.g., from noise, ototoxic compounds, or inflammatory processes, form part of the underlying genetically determined variation in risk for and development of human age-related hearing loss.

Associations linked to *SYNJ2* and *GRM5* point towards the cochlear conductance of auditory signals to play a role in hearing loss risk. Both genes encode components of neuronal signaling: *GRM5* encodes the metabolic glutamatergic receptor 5, an excitatory G protein-coupled receptor that is activated by glutamate in the synaptic cleft, and *SYNJ2* encodes synaptojanin 2, which mediates presynaptic recovery of secretory vesicles after neurotransmitter release. Both proteins were also expressed in type I spiral ganglion neurons. SYNJ2 was also expressed in the outer spiral bundles, i.e., the synaptic connections between the auditory neurons and the outer hair cells.

Our findings also suggest that genetic variants that affect cochlear development and maintenance of cellular identity also contribute to the risk for age-related hearing loss. LMX1A and EYA4 are both transcriptional regulators that have been linked to the development and maintenance of cochlear structures. Our imaging reveals expression of both proteins in discrete structures of the adult human cochlea: within the spiral ganglion neuronal cells, EYA4 within cells of the epithelial layer that faces the *scala media*, and LMX1A in the organ of Corti. This suggests a role for these proteins in maintenance of the cellular state of these structures in the adult human cochlea.

### Limitations

The current study is limited by the lack of audiometric data collected in a standardized manner, which would allow for a more optimal assessment of hearing loss in study participants. Collection of such data require specialized equipment such as a sound-proof room, tone generator, and a trained audiologist for accurate measurements, and thus become difficult to perform on the scale that is commonly required for an adequately powered GWAS for complex traits. Self-report data are valuable proxies for hearing loss as the collection of these data is easily scaled. The large number of biologically relevant findings from GWAS, as determined from literature analyses and immunohistochemical staining of human cochlea, also serve as a validation of the utility of self-report data on hearing-related traits for large-scale studies. However, the type of hearing loss that UK Biobank participants suffer from cannot be determined from questionnaire data alone. Although age-related hearing loss can be considered the most common form of sensorineural hearing loss in the elderly (> 60 years) [153], other types of hearing loss are common at middle age, such as noise-induced hearing loss and hearing loss due to viral or bacterial infections or ototoxic medications, as well as otosclerosis. The presence of other types of hearing loss may lead to decreased statistical power due to etiological heterogeneities in the studied phenotypes. The results from the GWAS would therefore benefit from replication in additional cohorts with more well-characterized patients.

We are also unable to elucidate the genomic mechanisms that underlie the associations observed in the GWAS. We have instead used a bioinformatic approach to generate plausible mechanisms for associated loci and to select candidate genes for downstream experiments. For hearing-related traits, many associations were found at genetic loci that contain genes with established links to hearing, e.g., at human autosomal nonsyndromic deafness loci, or near genes that affect cochlear morphology in other species. We consider this type of overlap as strong evidence for these genes to be involved in the causal mechanisms that underlie the association with hearing loss. In vitro and in vivo modeling of the effects of genetic variants would allow for determining the causal variant or variants and provide more exact genomic models for how they affect hearing loss risk.

Our study also does not fully account for genetic effects that may differ between sexes or are specifically expressed only in females or males. A recent study on genetic data from the UK Biobank demonstrated that sex-specific effects in the genetic architecture can lead to issues when analyses are performed on a combined set of male and female participants, such as genetic effects being masked from detection due to differences in effect sizes or directions between sexes [154]. This was most prominent for genetic variants that are associated with distribution of body fat and certain inflammatory disease such as gout and ankylosing spondylitis and the study did not report differences in genetic effects for clinically diagnosed hearing loss (data field 41270: ICD10-diagnoses - H91 Other hearing loss (*n* = 10,175) and ICD10-H90 Conductive and sensorineural hearing loss (*n* = 2927)). Sex-specific effects may nonetheless be present for hearing loss-related traits, which can potentially be identified in stratified analyses.

### Conclusion

In conclusion, GWAS for hearing loss-related traits reveal a genetic architecture for hearing loss in humans. Imaging of GWAS-identified leads findings provide evidence that implicate processes of the spiral ganglion neuronal cells, such as neuronal plasticity and recovery after trauma, to be critical for hearing loss risk. The results also highlight the importance of structural components of the hair cells and hair cell stereocilia and suggest that structural resilience within the organ of Corti is an important factor in determining the risk for sensorineural hearing loss. Importantly, proteins were selected for imaging based on previous cochlear imaging studies in other species. Consequently, it is essential to consider other aspects related to cochlear function that may also be influenced by common hearing loss-associated genetic variation. These findings provide biological insight of the underlying mechanisms of age-related hearing loss among the general population and also offer interesting leads for the development of potential novel therapeutic targets.

## Methods

### Study cohort

This research has been conducted using the UK Biobank resource under application number 26808. The UK Biobank is a large cross-sectional cohort of almost half a million participants from the UK that were recruited between 2006 and 2010 [155]. Participants were assessed at 22 centers across the UK where they provided biological samples and answers to an extensive touchscreen questionnaire and underwent physical examinations. At assessment, participants were between aged 37 and 73 years. The cohort was filtered for participants who self-identified as Caucasian and British and were identified as Caucasian by genetic principal component analysis, to avoid eventual issues caused by population stratification. Participants were also filtered to remove participants that exhibited high genetic relatedness (i.e., closely related), poor genotype call rate (< 95%), and high heterogeneity. Participants with discrepancies between genetic and self-reported sex, or a high rate of missing genetic markers were also removed. After filtering, 362,396 participants remained for analyses.

### Phenotypes

Hearing aid use, hearing difficulty, and tinnitus were assessed via touch-screen questionnaire. Participants were asked “Do you use a hearing aid most of the time?” and could answer “Yes” or “No” (data field 3393). For hearing difficulty, participants were asked “Do you have any difficulty with your hearing?” and could respond “Yes,” “No,” or “I am completely deaf” (data field 2247). For tinnitus, participants were asked “Do you get or have you had noises (such as ringing or buzzing) in your head or in one or both ears that lasts for more than 5 min at a time?,” and could answer “Yes, now most or all of the time,” “Yes, now a lot of the time,” “Yes, now some of the time,” “Yes, but not now, but have in the past,” or “No, never” (data field 4803). Hearing aid use and hearing difficulty responses were coded as 1 (“Yes”) or 0 (“No”). Participants who declined to answer or answered “I am completely deaf” were set as missing. For tinnitus, “Yes, now most or all of the time,” “Yes, now a lot of the time,” “Yes, now some of the time,” or “Yes, but not now, but have in the past” were coded as 1 and “No, never” was set as 0.

Speech-in-noise perception was assessed during the touchscreen questionnaire session. The participant was supplied headphones and assessed for the ability to hear three spoken digits alongside a rushing background noise [156]. Participants’ left and right ears were tested separately. For each ear, fifteen triplets of numerical digits along with background noise are presented to the participant. The signal-to-noise ratio ranges from − 12 dB to + 8 dB. The test aims to establish the speech reception threshold, i.e., the signal-to-noise ratio at which the participant is able to comprehend half of the spoken information. It accomplishes this by raising the noise level after a correct response, and lowering it after an incorrect one. The speech recognition threshold for each ear correspond to the value of the signal-to-noise ratio at the last round of the test for participants who completed all 15 rounds [157] and is provided for the left and right ear (data fields 20019 and 20021). Speech-in-noise was recoded as the mean speech recognition threshold for both ears. Mean thresholds were rank transformed using the “rntransform” function, which is included in the GenABEL package [158], in R [159] while adjusting for age and age-squared.

### Genotyping

UK Biobank participants were genotyped on two separate microarrays. Initially, approximately 50,000 participants were genotyped on the Affymetrix UK BiLEVE Axiom array as part of the UK BiLEVE (UK Biobank Lung Exome Variant Evaluation) study [160]. Approximately 450,000 participants were subsequently genotyped on the Affymetrix UK Biobank Axiom array. The two microarrays each contain probes for 807,411 and 825,927 markers, respectively, with 95% overlap of marker content between arrays [161]. Imputation predicts the genotypes that were not directly assayed in a population. This is achieved by utilizing densely genotyped data sets as reference. In the UK Biobank, imputation of up to 92,693,895 SNPs, insertion-deletions, and large structural variants was carried out using a merged reference set from UK10K, 1000 genomes, and the haplotype reference consortium [161, 162].

### Genome-wide association study, GWAS

Genome-wide association tests were performed with Plink v1.90b3n [116]. Hearing aid use, hearing difficulty, and tinnitus were analyzed by logistic regression modeling, and speech-in-noise was analyzed by linear regression modeling. Models included sex and age as covariates, as well as a batch variable to adjust for the genotyping platform (Affymetrix UK BiLEVE Axiom array or Affymetrix UK Biobank Axiom array). Models also included the first 15 genetic principal components that were provided by UK Biobank to adjust for any residual effects of population stratification. Genotypes were filtered for minor allele frequency (MAF > 0.01%), genotype call rate (> 95%), and deviation from Hardy-Weinberg equilibrium (*P* < 1 × 10^−20^). This left approximately 35 million SNPs for association testing. Association analyses were performed on the computational cluster at the Uppsala multidisciplinary center for advanced computational science (UPPMAX) under project sens2017538.

### Clumping

Genome-wide association analyses results in a large number of associations between genetic variants and the interrogated phenotype. Associations are commonly seen for many SNPs in close proximity of each other, due to the linkage disequilibrium between SNPs. To determine the number of independent associations, we perform “clumping,” where associated SNPs at each locus are clumped together, and an independent “lead SNP” is identified. Clumping is included as a function in Plink v1.90b3n under the --clump flag and was performed for all traits. We set a lenient threshold for genome-wide significance in the clumping analyses: “--clump-p1 5 × 10^−7^” for the lead SNP. We used a lenient threshold for correlation between SNPs, “--clump-r2 0.1”, and “--clump-kb 500,” so that all SNPs within 500 kb with a squared correlation of *R*^*2*^ > 0.1 to the lead SNP were assigned to the clump that was represented by the lead SNP. A secondary significance threshold for clumped SNPs of “--clump-p2 5 × 10^−7^” was also used to designate genome-wide significant SNPs within the clump. We also used a hg19 gene range list to cross-reference and report the overlap between clumps and genes, which is performed with the “--clump-range” flag.

### Linkage disequilibrium (LD) score regression

LD score regression (LDSC, v.1.0.0) [163] was used to generate heritability estimates, genomic inflation factors *(λ*_*GC*_*)*, mean *χ*^2^ statistics, and LD-score regression intercepts for each GWAS. LD scores from a random subset of 5000 UK Biobank participants were used as weights to adjust for correlation between SNPs. LD scores were generated from genotype data in the Plink -bed format with the LDSC software using a 1000-kb window around each SNP. Mean *χ*^2^ represents the mean of the *χ*^2^ statistics for all variants that were tested for association in the GWAS. One minus the intercept of the *χ*^2^ statistics regressed against the LD score (LD score regression) provides an estimate of the mean contribution of the confounding bias in the test statistic. This can be presented as a ratio or the proportion of the *χ*^2^ statistic that the LD score regression intercept ascribes to causes other than polygenic heritability (Ratio = LD intercept − 1)/(mean(*χ*^2^) − 1). In practice, the ratio is commonly around 10–20% [163]. The GWAS summary statistics can be adjusted for inflation by dividing the *t*-statistic with the genomic inflation factor and recalculating the *p* values.

### Cross reference of GWAS data with functional annotations from databases

To determine possible causal variants and link the associated loci in our GWAS to candidate genes, we cross-referenced our GWAS results with functional annotations from publicly available databases. Databases used were dbSNP build 150 (www.ncbi.nlm.nih.gov/snp) [164], the genotype tissue expression database (GTEx, gtexportal.org) [165], and the GWAS Catalog (https://www.ebi.ac.uk/gwas, dataset accessed 1 Mar 2019) [166]. dbSNP was filtered for non-synonymous missense, nonsense, and frameshift variants before cross-reference. Recent GWAS that had yet to be included in the GWAS Catalog were cross-referenced manually.

The linkage disequilibrium (LD) pattern for each lead SNP from our GWAS was determined using the “--r2” command with the flags “--ld-snp,” “--ld-window-r2 0.1,” and “--ld-window-kb 2000” in Plink v1.90b3n. All SNPs within a 2-Mb window around each lead SNP that were in linkage disequilibrium with the lead SNP (*R*^*2*^ > 0.1) were then identified and annotations for all SNPs were extracted from dbSNP and the GWAS catalog. SIFT and PolyPhen2 scores for nonsense, missense, and frameshift mutations were identified manually from Ensembl (www.ensembl.org). SIFT and PolyPhen are tools for predicting the functional consequences of genetic variants within protein-coding regions based on sequence homology as well as the physicochemical properties between the alternate amino acids [109, 110].

For the GTEx database, we determined the correlation of the lead SNPs from our analyses with expression quantitative trait loci (eQTLs) that were included in GTEx. We used a significance threshold for association of genetic variants with gene expression in GTEx of *P* < 2.3 × 10^−9^, in line with previous studies [167]. The correlations between the lead SNPs from our GWAS analyses and eQTLs from GTEx were determined and genetic variants in LD (*R*^*2*^ > 0.8) were considered to overlap.

### Literature review

We wished to determine if any genes within our GWAS-identified loci had been previously linked to hearing or hearing-related traits in humans or other species. We therefore performed a PubMed search of all genes within the clumps that were determined by Plink combined with the terms “AND Hearing.” The search results were then manually filtered for original peer-reviewed research articles that had reported data on at least one of the genes included in the literature search.

### Collecting and processing human tissue

The surgical specimens were from patients suffering from life-threatening posterior cranial fossa meningioma compressing the brain stem. The operations were performed at Uppsala University Hospital by a team of neurosurgeons and oto-neurosurgeons. Human cochleae were dissected out using diamond drills of various sizes. Tissues were immediately placed in 4% paraformaldehyde diluted with 0.1 M phosphate-buffered saline (PBS; pH 7.4) in the operating room. After a 24-h period spent in fixative, specimens were washed in 0.1 M PBS and then placed in 10% Na-EDTA solution at pH 7.2 for decalcification. The Na-EDTA solution was changed every 2 days until the decalcification process was completed, which took approximately 3 to 6 weeks. Decalcified cochleae were rinsed with 0.1 M PBS and placed in 25% sucrose in 0.1 M PBS overnight (4 °C). Cochleae were embedded in Tissue-Tek (OCT Polysciences) for frozen sections. Cochleae were rapidly frozen and sectioned at 8–10 μm using a Leica cryostat microtome. Frozen sections were collected onto gelatin/chrome-alum-coated slides and stored in a freezer of − 70 °C before immunohistochemistry procedures [168, 169]. In total, five specimens were available for staining. Patient characteristics are presented in Additional file 1: Table 7.

### Antibodies and immunostaining procedures

Immunohistochemistry procedures on human cochlear sections were described in previous publications [170, 171]. Sections were incubated with an antibody solution under a humidified atmosphere at 4 °C for 20 h. Sections were incubated with secondary antibodies conjugated to Alexa Fluor (Thermo Fisher Scientific, Uppsala) counterstained with the nuclear stain 4′,6-diamidino-2-phenylindole dihydro-chloride (DAPI), mounted with ProLong® Gold Antifade Mountant (Thermo Fisher Scientific, Uppsala, Catalog number: P10144), and then covered with the specified cover glass compatible with both confocal and super-resolution microscopes. Primary and secondary antibody controls and labeling controls were used to exclude endogenous labeling or reaction products [172]. The antibodies used for immunohistochemistry are shown in Additional file 1: Table 8.

### Imaging and photography

Stained sections were first investigated with an inverted fluorescence microscope (TE2000; Nikon, Tokyo, Japan) equipped with a spot digital camera with three filters (for emission spectra maxima at 358, 461, and 555 nm). Image-processing software (NIS Element BR-3.2; Nikon), including image merging and a fluorescence intensity analyzer, was installed on a computer system connected to the microscope. For laser confocal microscopy, we used the same microscope equipped with a three-channel laser emission system. SR-SIM [173] was performed using a Zeiss Elyra S.1 SIM system and a × 63/1.4 oil Plan-Apochromat objective (Zeiss, Oberkochen, Germany), sCMOS camera (PCO Edge), and ZEN 2012 software (Carl Zeiss Microscope). The resolution of the SR-SIM system at BioVis, Uppsala University, was 107 nm in the *X*–*Y* plane and 394 nm in the *Z* plane. The laser and filter were set up as follows: 405 nm laser of excitation coupled with BP 420–480 + LP 750 filter, 488 nm laser of excitation with BP 495–550 + LP750 filter, 561 nm laser of excitation with BP 570–620 + LP 750 filter, and 647 nm laser of excitation with LP 655 filter. From the SR-SIM dataset, 3D reconstruction was done with Imaris 8.2 (Bitplane, Zürich, Switzerland). A bright-field channel was able to merge fluorescence channels to visualize cell and tissue borders.

## Supplementary Information

**Additional file 1: Figures 1-3**, **Tables 1-8**. **Fig 1.** Manhattan plots that illustrate the results from genome-wide association analyses for hearing difficulty, hearing aid use, speech-in-noise and tinnitus. **Fig 2.** Quantile-quantile plots for the results from genome wide association analyses on hearing difficulty, hearing aid use, speech-in-noise and tinnitus. **Fig 3.** Comparison of results from the current study and the recent GWAS for hearing difficulty. **Table 1.** Characteristics of UK Biobank participants that were included in GWAS for hearing-related traits. **Table 2.** Results from the LDSC analyses on hearing loss-related trait GWAS in the UK Biobank. **Table 3.** Brief description of the literature for genes within genetic loci that were associated with hearing-related traits in the UK Biobank. **Table 4.** Non-synonymous coding variants that were linked to genetic loci associated with hearing-related traits in the UK Biobank. **Table 5.** Expression quantitative trait loci (eQTLs) that were found to be linked to genetic loci associated with hearing related traits in the UK Biobank. **Table 6.** Summary of expression patterns of candidate proteins in other mammals. **Table 7.** Characteristics of tissue donors. **Table 8.** Antibodies used for immunohistochemical staining.

**Additional file 2: **Excel sheets with the lead SNPs that were associated with hearing difficulty, hearing aid use, speech-in-noise and tinnitus. Columns: CHR = chromosome, SNP = variant ids, either as rsID or in the format CHR_POSITION_ALLELE1_ALLELE2, BP = chromosomal position in basepairs (Genome Reference Consortium Human Build 37, GRCh37), *P* = P-value for association tests, P.GC_adjusted = P-values after adjusting for genomic inflation (this was only performed for tinnitus), SIGNIFICANT_ASSOCIATION = denotes whether a genome-wide association could be observed after adjusting for the family-wise error rate with Bonferroni-correction (*P* < 5*10^-8^), SNPS_IN_CLUMP (N) = the number of associated SNPs that were in linkage disequilibrium with the lead SNPs, CLUMP_COORDS = coordinates for the first and last associated SNPs that were clumped with the lead SNP, CLUMP_SIZE (bp) = the size of the clumped region in basepairs, GENES_IN_CLUMP = genes that were located within each clumped region.

## Data Availability

GWAS summary statistics are publically available from GWAS Catalog (https://www.ebi.ac.uk/gwas/) [174].

## Abbreviations

bas.: Basilar membrane
BTF: Basal tunnel fibers
Cl: Cells of Claudius
cut.: Cuticula
DAPI: 4′,6-Diamidino-2-phenylindole dihydrochloride
dbSNP: The Single Nucleotide Polymorphism Database
DC: Deiters cell
eff: Tunnel spiral bundle
eQTLs: Expression quantitative trait loci
GTEx: Genotype-Tissue Expression project
GWAS: Genome-wide association study
HC: Hensen cell
ICs: Inderdental cells
IHC: Inner hair cell
IPC: Inner pillar cell
IS: Inner sulcus
kb: Kilo basepairs (i.e., 1000 base pairs)
LD: Linkage disequilibrium
LDSC: LD score regression
MAF: Minor allele frequency
MNTB: Medial nucleus of the trapezoid body
*n*: Afferent/efferent neurons
Na-EDTA: Sodium-ethylene-diamine-tetra-acetic acid
OC: Organ of Corti
OHC: Outer hair cell
OHCs: Outer hair cells
OPC: Outer pillar cell
OS: Outer sulcus
OSBs: Outer spiral bundles
PBS: Phosphate buffered saline
Ph: Phalangeal cells
pH: The base 10 logarithm of the reciprocal of the hydrogen ion activity in a solution
PiC: Pillar cell
PolyPhen2: Polymorphism Phenotyping version 2
Pv: Parvalbumin
*r*: Stereociliar rootlet
RL: Reticular lamina
RNA: Ribonucleic acid
SC: Stereocilia
SIFT: Sorting Intolerant From Tolerant missense variant prediction tool
SNPs: Single-nucleotide polymorphisms
SP: Spiral prominence
SR-SIM: Super-resolution Structured Illumination Microscopy
T1: Type I spiral ganglion cells, alternatively, large spiral ganglion cells
TJ: Tight junction
TSB: Tunnel spiral bundle
TUJ1: Neuron-specific class III beta-tubulin
UK BiLEVE: UK Biobank Lung Exome Variant Evaluation
UK: United Kingdom
UPPMAX: Uppsala multidisciplinary center for advanced computational science
*λ*_*GC*_: Genomic inflation factor
