# Supplementary material for: A combined genome-wide association and molecular study of age-related hearing loss in H. sapiens
Source: BMC Med. 2021 Dec 1;19:302. doi: 10.1186/s12916-021-02169-0 (PMC8638543; doi:10.1186/s12916-021-02169-0)
Supplement: Supplementary file 1 — Additional file 1: Figures 1-3, Tables 1-8. Fig 1. Manhattan plots that illustrate the results from genome-wide association analyses for hearing difficulty, hearing aid use, speech-in-noise and tinnitus. Fig 2. Quantile-quantile plots for the results from genome wide association analyses on hearing difficulty, hearing aid use, speech-in-noise and tinnitus. Fig 3. Comparison of results from the current study and the recent GWAS for hearing difficulty. Table 1. Characteristics of UK Biobank participants that were included in GWAS for hearing-related traits. Table 2. Results from the LDSC analyses on hearing loss-related trait GWAS in the UK Biobank. Table 3. Brief description of the literature for genes within genetic loci that were associated with hearing-related traits in the UK Biobank. Table 4. Non-synonymous coding variants that were linked to genetic loci associated with hearing-related traits in the UK Biobank. Table 5. Expression quantitative trait loci (eQTLs) that were found to be linked to genetic loci associated with hearing related traits in the UK Biobank. Table 6. Summary of expression patterns of candidate proteins in other mammals. Table 7. Characteristics of tissue donors. Table 8. Antibodies used for immunohistochemical staining. [file 12916_2021_2169_MOESM1_ESM.docx]

**Table 1.** Characteristics of UK Biobank participants that were included in GWAS for hearing-related traits

|  | **Total** | **Females** | **Males** | **Difference**  **(P)** |
| --- | --- | --- | --- | --- |
| UK Biobank participants included in study (N) | 362396 | 195008 | 167388 |  |
| Percentage female and male participants |  | 53.8% | 46.2% |  |
| Mean age (years)^*^ | 56.9 | 56.7 | 57.1 | <2.2*10^-16^ |
| Age standard deviation (years) | 8.0 | 7.9 | 8.1 |  |
| Reported difficulty hearing  (data field 2247, N, yes/no)^**^ | 91,080/257,037 | 40,244/146,172 | 50,836/110,865 | <5.0*10^-303^ |
| Reported difficulty hearing (%) | 26.2% | 21.6% | 31.4% |  |
| Reported use of hearing aid  (data field 3393, N, yes/no)^**^ | 11,081/208,083 | 4900/105,416 | 6181/102,667 | 7.0*10^-40^ |
| Reported use of hearing aid (%) | 5.1% | 4.4% | 5.7% |  |
| Reported having or ever having tinnitus  (data field 4803, N, yes/no)^**^ | 34,091/83,069 | 16,525/46,228 | 17,566/36,841 | 7.0*10^-111^ |
| Reported having or ever having tinnitus (%) | 29.1% | 26.3% | 32.3% |  |
| Participated in speech reception threshold (speech-in-noise) testing (N, yes/no)^**^ | 110,717/251,679 | 59,348/135,660 | 51,369/116,019 | 0.10 |
| Participated in speech reception threshold (speech-in-noise) testing (%) | 30.6% | 30.4% | 30.7% |  |
| Mean average speech recognition threshold (left and right thresholds divided by two)^*^ | -6.67 | -6.69 | -6.64 | 9*10^-9^ |
| Standard deviation, average speech recognition threshold (left and right thresholds divided by two) | 1.64 | 1.55 | 1.72 |  |
| Mean speech reception threshold (speech in noise), left ear (data field 20019)^*^ | -6.58 | -6.61 | -6.54 | 3.1*10^-8^ |
| Standard deviation, speech reception threshold (speech in noise), left ear (data field 20019) | 1.94 | 1.85 | 2.04 |  |
| Mean speech reception threshold (speech in noise), right ear (data field 20021) | -6.54 | -6.57 | -6.50 | 1.0*10^-8^ |
| Standard deviation, speech reception threshold (speech in noise), right ear (data field 20021) | 1.95 | 1.86 | 2.05 |  |

^*^The Student’s t-test was used to test for differences in continuous variables.

^**^Chi-squared tests were used to test for differences in prevalence of hearing-loss associated traits between males and females

**
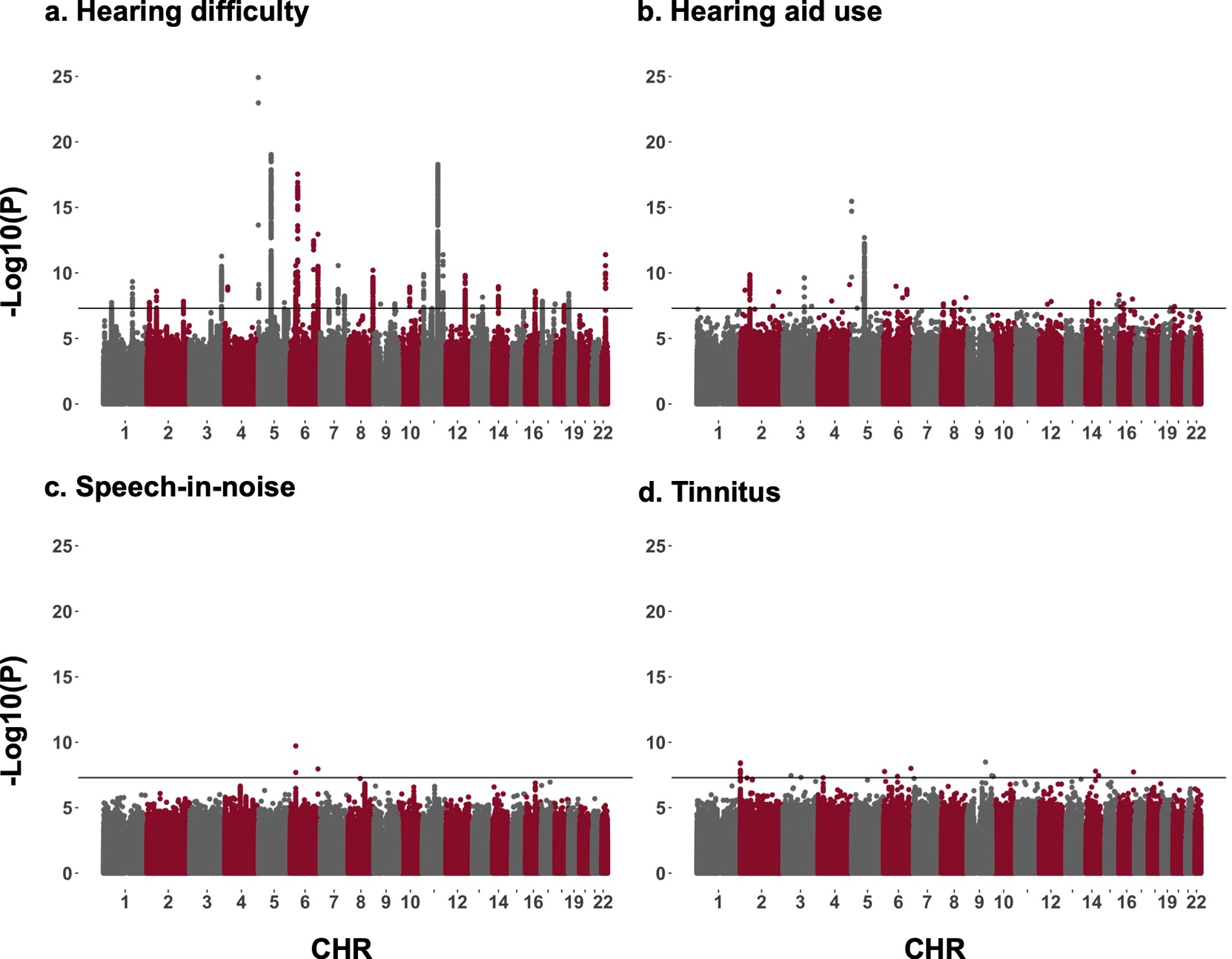
**

**Fig 1.** Manhattan plots that illustrate the results from genome-wide association analyses for a) hearing difficulty, b) hearing aid use, c) speech-in-noise and d) tinnitus in the UK Biobank.


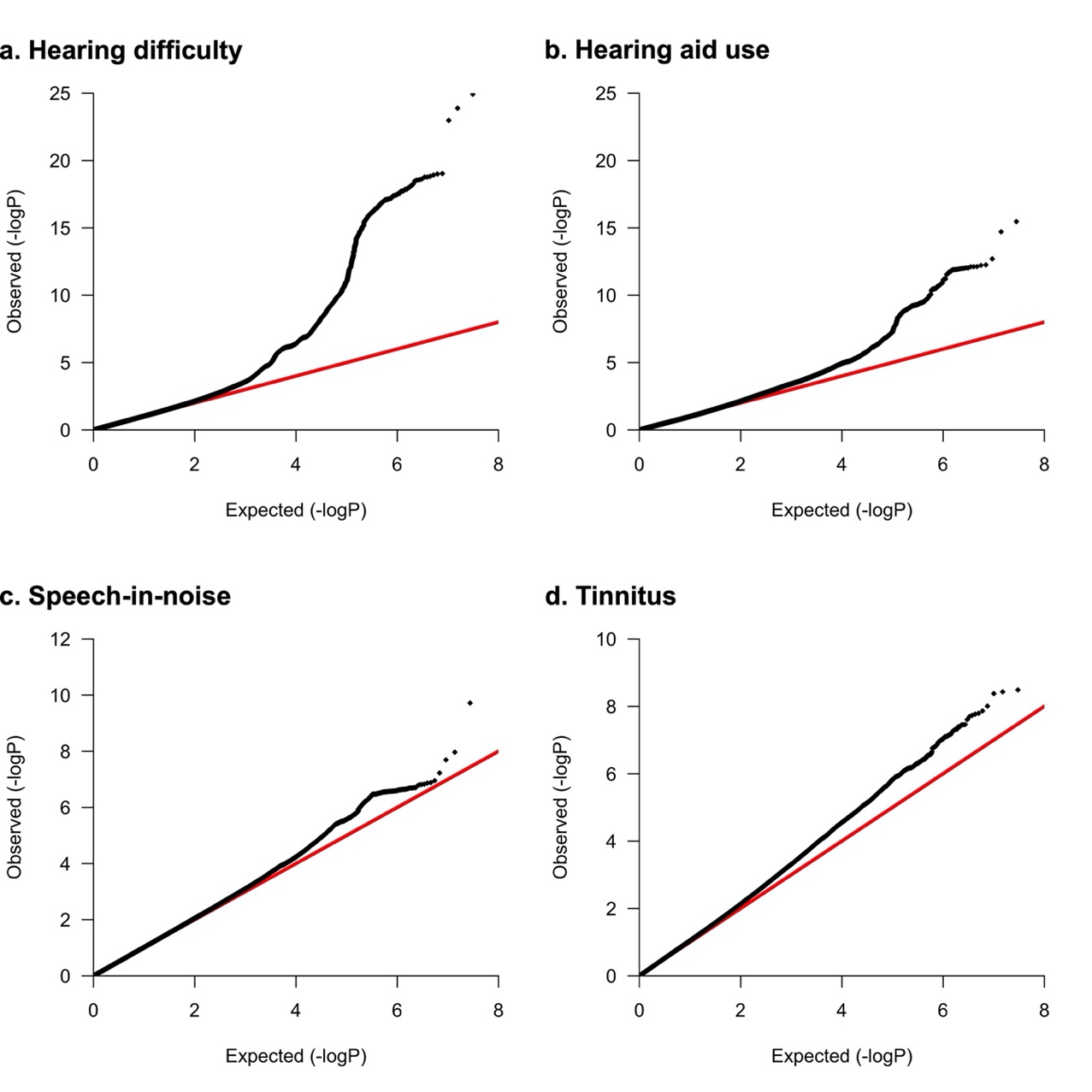


**Fig 2.** Quantile-quantile plots for the results from genome wide association analyses on a) hearing difficulty, b) hearing aid use, c) speech-in-noise and d) tinnitus in the UK Biobank.

**Table 2. Results from the LDSC analyses on hearing loss-related trait GWAS in the UK Biobank.**

|  | Hearing difficulty | Hearing aid use | Speech-in-noise | Tinnitus |
| --- | --- | --- | --- | --- |
| Total observed scale h^2^ | 3.85 ± 0.45% | 0.94 ± 0.27% | 3.01 ± 0.98% | 1.26 ± 0.29% |
| λ_GC_ | 1.24 | 1.07 | 1.08 | 1.17 |
| Mean χ^2^ | 1.31 | 1.09 | 1.09 | 1.17 |
| LD regression intercept | 1.03 ± 0.02 | 1.02 ± 0.01 | 1.02 ± 0.01 | 1.08 ± 0.02 |
| Ratio | 11 ± 5% | 21 ± 15% | 23 ± 16% | 46 ± 9% |

Observed scale heritability *(h^2^)* corresponds to the proportion of the variance in the trait that can be attributed to genetic factors. *λ_GC_* is the genomic control factor. Mean *χ^2^* represents the mean of the *χ^2^* statistics for all variants that were tested for association in the GWAS. One minus the intercept of the *χ^2^* statistics regressed against the LD score (LD score regression) provides an estimate of the mean contribution of the confounding bias in the test statistic. This can be presented as a ratio or the proportion of the *χ^2^* statistic that the LD score regression intercept ascribes to causes other than polygenic heritability (Ratio = LD intercept-1)/(mean*(χ^2^)*-1).

**Table 3.** Brief description of the literature for genes within genetic loci that were associated with hearing-related traits in the UK Biobank.

| Lead SNP | Chr | Position (bps) | phenotype | Candidate gene(s) | Non-syndromic deafness locus* | Brief description of previously published literature | |  |  |
| --- | --- | --- | --- | --- | --- | --- | --- | --- | --- |
| rs1806319 | 11 | 88,543,715 - 89,162,175 | Hearing difficulty | *NOX4, TYR* |  | *TYR* encodes tyrosinase, which catalyses the rate-limiting step in production of melanin and is expressed in melanocytes. Melanocytes are distributed in the human cochlea [10] and melanin or its precursors has been suggested to protect against age-related or noise-induced hearing loss, potentially by acting as a scavenger of reactive oxygen species (ROS) [11]. *NOX4* encodes NADPH oxidase 4, which is a constitutively active producer of ROS. Studies in NOX4-transgenic mice revealed higher vulnerability to noise-induced hearing loss [12]. | |  |  |
| rs9493627 | 6 | 133,789,728 - 133,836,188 | Hearing difficulty, Hearing aid | *EYA4* | DFNA10 [13] | Mutations in *EYA4* have been frequently associated with late-onset hearing loss [13, 14], hearing impairment [15], dilated cardiomyopathy and sensorineural hearing loss [16], nonsyndromic hearing loss [17–23], autosomal dominant middle-frequency sensorineural hearing loss [24], noise induced hearing loss [25–28], moderate degree hearing loss [29], post-lingual hearing loss [30], sensorineural hearing loss and mild cardiac phenotype [31]. *EYA4* functions as a histone phosphatase and has been suggested to play a role in organogenesis (UniProt). Cochlear expression of *EYA4* was mapped in the common marmoset (*Callithrix jacchus*) and showed strong expression in supporting cells, which was diminished in mice [32]. | |  |  |
| rs739138 | 22 | 38,116,507 - 38,157,805 | Hearing difficulty | *TRIOBP* | DFNB28 [33, 34] | *TRIOBP* has previously been linked to the DFNB28 deafness locus which is associated with recessively inherited, prelingual, profound sensorineural hearing loss [34]. Studies have repeatedly identified rare mutations in *TRIOBP* to be associated with autosomal recessive non-syndromic hearing loss [35–37], sporadic non-syndromic hearing loss [38], congenital moderate hearing loss [39, 40] and non-syndromic deafness [41]. TRIOBP is an actin-bundling protein that locates to the stereocilia rootlets in mice, a supportive paracrystalline array of actin filaments that extend from the base of the stereocilia into the hair cell body. Transgenic mice with *Triobp* defects fail to develop stereocilia rootlets and are deaf [42]. | |  |  |
| rs141952919 | 7 | 102,590,946 - 103,061,825 | Hearing difficulty | *SLC26A5* | DFNB61 [9] | *SLC26A5* encodes the canonical hearing protein prestin, the motor protein of the outer hair cells, which enables amplification of incoming sound via movements of the outer hair cells in response to electrical stimulation, i.e. electromotility [43]. Variants that affect SLC26A5 are implicated as causal for the autosomal recessive deafness locus DFNB61. Prestin has been intensely studied in relation to hearing. Querying PubMed for "SLC26A5 AND HEARING" yields more than a hundred entries. | |  |  |
| rs138501510 | 6 | 158,471,260 - 158,599,382 | Hearing difficulty, Speech-in-noise | *SERAC1, SYNJ2* |  | In mice, *Synj2* is expressed in the organ of Corti, in the inner and outer hair cells [44]. The Mozart mouse strain, which carries a *Synj2* mutation, are born with normal hearing that deteriorates by eight leading to deafness by twelve weeks. Mozart mice also exhibit hair cell degeneration with fusion of stereocilia followed by loss of hair bundles and subsequent loss of hair cells [44]. *SYNJ2* encodes the lipid phosphatase synaptojanin 2, which removes the 5-position phosphate from phosphoinositides such as PIP3 and PIP2. Mutations within *SERAC1* causes MEGDEL syndrome (3-methylglutaconic acuduria, dystonia-deafness, hepatopathy, encephalopathy, Leigh-like syndrome) [45–49]. | | |  |
| rs1962104 | 8 | 141,604,684 - 141,932,130 | Hearing difficulty | *AGO2, PTK2/FAK* |  | *AGO2* encodes argonaut 2, which is involved in RNA mediated gene-silencing. AGO2 mRNA was observed to be upregulated in whole blood samples of 37 patients with sensorineural hearing loss [50]. *PTK2* encodes the focal adhesion kinase (FAK), which localizes to the mouse apical hair-cell surface where stereocilia are forming together with integrin α8β1 and fibronectin [51]. FAK is a non-receptor protein-tyrosine kinase that regulates cellular adhesion and mobility. | |  |  |
| rs55635402 | 11 | 8,053,304 - 8,085,652 | Hearing difficulty | *TUB* |  | The 'tubby' mouse phenotype, which is characterized by maturity-onset obesity, insulin resistance and sensory deficits, is caused by a mutation in the *Tub* gene [52]. Tubby mice suffer sensorineural hearing loss [53, 54]. | | |  |
| rs1558804 | 12 | 109,788,598 - 110,042,348 | Hearing difficulty | *UBE3B* |  | Recessive mutations in *UBE3B* are causal for Kaufman oculo-cerebro-facial syndrome, which has been linked to hearing loss [55, 56]. In addition, *UBE3B* has been found to be highly expressed following noise trauma in the chick basilar papilla [57]. *UBE3B* was suggested as a causal gene for the DFNA25 locus [58, 59], which was subsequently attributed to mutations in *SLC17A8* [60]. | | |  |
| rs6941393 | 6 | 32,454,348 - 32,607,906 | Speech-in-noise, Hearing difficulty | *HLA-DQA1, HLA-DRB1, HLA-DRB5* |  | Different alleles of the HLA class II histocompatibility antigens DRB1 and DQA1 have been associated with Ménière's disease (MD), sudden onset sensorineural hearing loss (SNHL) as well as a differential response to steroid treatment for SNHL: The HLA-DRB1*1101 allele has been associated with MD in a Mediterranean population from Spain [61], while the HLA-DRB1*15 has been associated with MD in a sample of South Korean patients . The HLA-DRB1*0403 allele has been associated with SNHL in a sample of patients from Galicia, northern Spain [62]. The HLA-DRB1*14 allele was associated with SNHL in a sample of patients from Seoul, South Korea while HLA-DRB*04 was associated with lack of response to corticosteroid treatment [63]. Response to corticosteroid treatment for SNHL has also been associated with the HLA-DRB1*14, HLA-DQA1*03, *05 and *01 alleles [64]. HLA-DRB1*0405 was also present in a case report of sudden onset of blindness, pleocytosis, and temporary Hearing Loss in a 47-year-old man with Vogt-Koyanagi-Harada syndrome [65]. | |  |  |
| rs36062310 | 22 | 50,950,570 - 51,146,132 | Hearing difficulty, Hearing aid | *TYMP* |  | Mutations in TYMP are linked to mitochondrial neuro-gastrointestinal encephalomyopathy (MNGIE), a rare multisystemic disorders which involved hearing loss [66, 67]. | |  |  |
| rs7525101 | 1 | 165,087,254 - 165,112,224 | Hearing difficulty | *LMX1A* |  | *Lmx1a* mutations in mice have been linked to cochlear and vestibular defects along with severe hearing impairment [68]. *Lmx1a* regulates gene expression and cell fate and is involved in formation of the cochlear morphology and formation of the sensory epithelia of the organ of Corti in mammals [69]. | |  |  |
| rs2393729 | 10 | 63,828,879 - 63,841,130 | Hearing difficulty | *ARID5B* |  | Cochlear *Arid5b* is elevated in aging C57BL/6 mice, which display early onset age-related hearing loss. Arid5b is a transcriptional regulator that was observed to regulate the long non-coding RNA AW112010, which was increased in aging C57BL/6 mouse cochleae [70]. Arid5b/AW112010 was shown to regulate mitochondrial function in HEI-OC1 cell and hypothesized to protect cochlear hair cells from oxidative stress [70]. | |  |  |
| rs78528263 | 16 | 55,474,324 - 55,507,592 | Hearing difficulty | *MMP2* |  | *MMP2* encodes the 72 kDa type IV collagenase (alternatively, matrix metalloproteinase 2), which cleaves the extracellular structure proteins gelatin type I and collagen types IV, V, VII, X. *Mmp2*, along with *Mmp9*, are increased in the guinea pig cochlea following acoustic noise trauma [71] and cochlear implantation [72]. *Mmp2* was found to be expressed in rat spiral ganglion neurons and higher *Mmp2* expression was observed after treatment with the ototoxic aminoglycoside antibiotic amikacin [73]. Topical administration of MMP inhibitors oxytetracycline and ilomastat showed a protective effect against LPS-induced cochlear lateral wall damage in guinea pigs [74]. *Mmp2* expression was also increased following acute otitis media following middle ear injection of heat-killed *Streptococcus pneumoniae* in Balb/c mice [75]. *Mmp2* was also observed to be upregulated in the cochlea of hyper-homocysteinemic cystathione beta-synthase heterozygous knockout mice [76]. | |  |  |
| rs2941580 | 2 | 54,728,276 - 54,966,407 | Hearing difficulty | *SPTBN1* | DFNA58 [77] | DFNA58 is an autosomal dominant deafness locus [77]. *SPTBN1* encodes the beta-II spectrin subunit. It is expressed in the cuticula of the inner and outer hair cells where they organize actin around the stereocilia rootlets [78]. Sptbn1 was observed to be preferentially expressed in the swim bladder of the channel catfish [79]. In catfishes, Weberian ossicles connect the swim bladder to the inner ear, which improves hearing ability. | |  |  |
| rs920701 | 13 | 76,343,051 - 76,455,056 | Hearing difficulty | LMO7 |  | Lmo7 is abundant in the chick cuticular plate and Lmo7 knock out mice display F-actin deficiencies in the cuticular plate, cochlear tuning and sensitivity abnormalities, as well as late-onset progressive hearing loss [80]. | |  |  |
| rs751007730 | 6 | 109,080,100 - 109,080,100 | Hearing aid | FOXO3 |  | Foxo3 encodes a transcription factor, which is expressed on the mouse cochlea [81]. It has been associated with noise-induced hearing loss in two studies on Chinese residents [82, 83]. Foxo3 knock-out mice develop adult-onset hearing loss due to auditory neuropathy, i.e. the hearing loss could not be attributed to hair cell loss or dysfunction but rather appears to be caused by disturbed synaptic function [81]. *Foxo3* was also found to be downregulated in Cytidine monophosphate-N-acetylneuraminic acid hydroxylase (*Cmah*) null mice, which exhibit age-related hearing loss [84]. In addition, cochlear *Foxo3* expression was also upregulated in beta-catenin overexpressing mice, which are protected against neomycin-induced hair cell damage [85]. Moderate noise-exposure also leads to severe hearing loss and outer hair cell death in *Foxo3* knockout mice [86]. | |  |  |
| rs183870062 | 6 | 156,398,495 - 156,398,495 | Tinnitus | *ARID1B* |  | ARID1B disruption has been implicated in syndromes related to deletions on chromosome 6q25 that include hearing loss as one of the symptoms [87, 88]. | |  |  |
| rs1806319 | 11 | 88,486,635 - 88,537,621 | Hearing difficulty | *GRM5* |  | GRM5 encodes the metabolic glutamate receptor 5, a glutamate-activated G protein-coupled receptor. In rats, the *Grm5* antagonist 2-methyl-6-(phenyl ethynyl)-pyridine (MPEP) was observed to block tetanus-induced acoustically-evoked potentials from the medial geniculate nucleus to the lateral nucleus of the amygdala [89]. MPEP also inhibited the extinction of the startle response in rats in response to a startling sound [90]. | |  |  |
| rs7355485 | 2 | 14,473,058 - 14,534,949 | Hearing. |  |  |  |  |  |  |
| rs7355485 | 2 | 14,473,058 - 14,534,949 | Hearing difficulty | *LINC00276* | DFNB47 [91] | DFNB47 is an autosomal recessive non-syndromic hearing loss locus that has been mapped to chromosome 2p24.3 [91, 92]. The GWAS locus is located in a gene desert, where *LINC00276* is the closest transcript at ~70kb upstream. Bilateral hearing impairment was also present, along with other symptoms, in a 26-month-old girl with a *de novo* 4.4 Mb microdeletion in chromosome 2p24.3-24.2 [93]. | |  |  |
| rs776705997 | 16 | 83,234,841 - 83,244,316 | Tinnitus | *CDH13* |  | CDH13 was implicated (P < 10^-7^) in a GWAS on hearing loss on 3417 Europeans [94]. Cdh13 was subsequently observed to be expressed in multiple cell types of the mouse cochlea: the inner and outer hair cells [95]. Rs17195859-AA within CDH13 was also associated with audiometric measurements of hearing loss [95]. | |  | |
| rs149212248 | 14 | 92,015,285 - 92,015,285 | Hearing aid | *CATSPERB* |  | *CATSPERB* encodes a component of the 'cation channels of sperm' protein complex. Genetic variants in the functionally related *CATSPER2* have been found to be causal for the deafness-infertility syndrome [96], and to be associated with hearing loss in a targeted sequencing study on candidate genes for hearing loss [38]. | |  | |
| rs143796236 | 17 | 79,495,969 - 79,524,724 | Hearing difficulty | *FSCN2* |  | *FSCN2* encodes the fascin 2 protein, which crosslinks actin to form filamentous bundles. A missense mutation in *Fscn2*, along with mutations in the stereocilia tip link protein cadherin 23, contribute to age-related hearing loss in DBA/2J mice. Fscn2 proteins are present in the retina and abundant in inner hair cell stereocilia, an concentrates near stereocilia tips [97]. Introduction of *Fscn2* mutations in C57BL/6J mice by genome editing with Transcription activator-like effector nuclease (TALEN) produced progressive hearing loss, along with degeneration of hair cells and loss of stereocilia [98]. *Fscn2* was also implicated in a mouse-GWAS for hearing loss [99]. *Fscn2* was also observed to be dynamically regulated during stereocilia growth [100]. |  |  |  |
| 9:140862253_C_A | 9 | 140,862,253 - 140,862,253 | Tinnitus | *CACNA1B, TPRN* | DFNB79  [97, 101] | DFNB79 is a nonsyndromic recessive deafness locus on chromosome 9q34.3 that spans 3.84Mb [101]. *TPRN* has been found to be causal for DFNB79 [97, 102]. This gene encodes a protein with unknown function. TPRN protein is expressed at the base of each stereocilia as they merge with the cuticula in outer and inner hair cells [97]. In CBA/CaJ mice, *Cacna1b* has been found to be expressed in spiral ganglion neurons [103] and to be downregulated following noise exposure [104]. CACNA1B, also known as, the voltage-gated calcium channel subunit alpha Cav2.2, encodes a subunit of the voltage-dependent N-type calcium channel, which gives rise to neuronal N-type currents. | |  |  |
| rs34859220 | 4 | 17,517,558 - 17,530,692 | Hearing difficulty | *CLRN2* |  | *CLRN2* encodes clarin-2, which appears to belong to a superfamily of integral four-transmembrane glycoproteins. It was recently identified as critical for hearing by genetic screening in mice [105]. *Clrn2* knock-out mice develop progressive, early onset hearing loss. Clrn2 is also expressed in the stereocilia of the inner and outer hair cells and is critical for maintaining stereocilia bundle integrity and function [106]. In addition, the paralog gene *Clrn1* has previously been shown to also be expressed in the hair cells and associated ganglionic neurons. *Clrn1-/-* mice also develop progressive early-onset hearing loss that leads to deafness [107]. | |  |  |
| rs17856487 | 22 | 38,485,540 - 38,487,526 | Hearing difficulty | *BAIAP2L2* |  | *Baiap2l2* is expressed in the hair cell bundle in mice [108]*.* *Baiap2l2* Deficient mice lose the 2nd and 3rd row of the inner- and outer hair cell stereocilia [108]. | |  |  |
| rs557019275 | 15 | 88,476,019 | Hearing aid use | *NTRK3* |  | *Ntrk3* was upregulated in spiral ganglion neurons after kainate-induced lesions in an *in vitro* model for auditory nerve regeneration in cochlear explants from P3-5 postnatal C57BL/6 mice [106]. | |  |  |

The lead SNP, chromosome and positions are included for each locus along with the associated trait, the candidate causal gene as determined by bioinformatic analyses and a review of the literature (see methods). A brief description of previous hearing-related findings is included for each locus. *Loci that overlap with non-syndromic deafness loci are annotated with the corresponding locus nomenclature. DFNA - autosomal dominant nonsyndromic deafness, DFNB - autosomal recessive nonsyndromic deafness.

**Table 4.** Non-synonymous coding variants that were linked to genetic loci associated with hearing-related traits in the UK Biobank.

| Lead SNP | Chr | Locus | Non-synonymous variant | Correlation with lead SNP *(R^2^)* | Gene name | Type | SIFT prediction* | SIFT score (range)* | PolyPhen-2 prediction** | PolyPhen-2 score (range)** |
| --- | --- | --- | --- | --- | --- | --- | --- | --- | --- | --- |
| rs139107036 | 3 | 45,942,584 - 45,942,584 | rs139107036 | 1.00 | *CCR9* | missense | deleterious | 0.00 | probably damaging | 1.00 |
| rs151025335 | 6 | 32,454,348 - 32,607,906 | rs1130382 | 0.68 | *HLA-DQB1* | missense | deleterious | 0.00 | probably damaging | 1.00 |
| rs6902016 | 6 | 158,471,260 - 158,599,382 | rs146694394 | 0.62 | *SYNJ2* | missense | deleterious | 0.00 | probably damaging | 1.00 |
| rs1806319 | 11 | 88,543,715 - 89,162,175 | rs1126809 | 0.72 | *TYR* | missense | deleterious | 0.03 | probably damaging | 0.99 |
| rs143796236 | 17 | 79,495,969 - 79,524,724 | rs143796236 | 1.00 | *FSCN2* | missense | deleterious | 0.00 | probably damaging | 0.97 - 0.99 |
| rs151025335 | 6 | 32,454,348 - 32,607,906 | rs1130381 | 0.68 | *HLA-DQB1* | missense | deleterious - tolerated | 0.02 - 0-07 | benign | 0.02- 0.15 |
| rs9493627 | 6 | 133,789,728 - 133,836,188 | rs9493627 | 1.00 | *EYA4* | missense | deleterious - tolerated | 0.00 - 0.22 | benign - probably damaging | 0.30 - 0.99 |
| rs17856487 | 22 | 38,485,540 - 38,487,526 | rs4289289 | 0.68 | *SLC16A8* | missense | deleterious - tolerated | 0.02 - 0.41 | benign | 0.00 - 0.30 |
| rs36062310 | 22 | 50,940,710 - 51,229,656 | rs36062310 | 1.00 | *KLHDC7B* | missense | deleterious - tolerated | 0.04 - 0.31 | benign - probably damaging | 0.04 - 1.00 |
| rs34859220 | 4 | 17,517,558 - 17,530,692 | rs13147559 | 0.99 | *CLRN2* | missense | tolerated | 0.06 | possibly damaging | 0.53 |
| rs36089056 | 1 | 46,027,355 - 46,598,273 | rs1707336 | 0.99 | *MAST2* | missense | tolerated | 0.37 - 0.65 | benign - probably damaging | 0.18 - 1.00 |
| rs2339607 | 5 | 72,922,376 - 73,198,901 | rs7714670 | 0.89 | *ARHGEF28* | missense | tolerated | 0.37 - 0.47 | benign | 0.00 |
| rs2339607 | 5 | 72,922,376 - 73,198,901 | rs6453022 | 0.75 | *ARHGEF28* | missense | tolerated | 0.29 - 0.67 | benign | 0.00 |
| rs553448379 | 6 | 43,260,660 - 43,408,005 | rs2242416 | 0.99 | *CRIP3* | missense | tolerated | 0.39-0.50 | benign | 0.34 - 0.05 |
| rs777434300 | 7 | 138,483,551 - 138,505,777 | rs61729750 | 0.82 | *TMEM213* | missense | tolerated | 0.66 - 1.00 | benign | 0.00 |
| rs1558804 | 12 | 109,788,598 - 110,042,348 | rs7298565 | 0.71 | *UBE3B* | missense | tolerated | 0.97 - 1.00 | benign | 0.00 |
| rs1558804 | 12 | 109,788,598 - 110,042,348 | rs9593 | 0.71 | *MMAB* | missense | tolerated | 0.10 - 1.00 | benign | 0.00 |
| rs920701 | 13 | 76,343,051 - 76,455,056 | rs7986131 | 0.63 | *LMO7* | missense | tolerated | 1.00 | benign | 0.00 |
| rs17671352 | 17 | 7,116,154 - 7,172,609 | rs4562 | 0.83 | *CLDN7* | missense | tolerated | 0.52 | benign | 0.00 |
| rs739138 | 22 | 38,116,507 - 38,157,805 | rs739138 | 1.00 | *TRIOBP* | missense | tolerated | 1.00 | benign | 0.00 |
| rs739138 | 22 | 38,116,507 - 38,157,805 | rs17856487 | 1.00 | *BAIAP2L2* | missense | tolerated | 0.45 - 0.50 | benign | 0.00 |

The lead SNP and coordinates are reported for each locus. The non-synonymous variant is reported along with its correlation with the lead SNP, the gene within which it is located, as well as SIFT [109] and PolyPhen-2 [110] prediction scores and qualitative descriptors as accessed from Ensembl.org. *SIFT performs prediction of functional consequences of genetic variants based on sequence homology [109]. For Ensembl, SIFT version 5.2.2 was run and the software was allowed to select homologous sequences by adding the most similar sequence from a database of protein sequences iteratively to the until conservation in the region of interest decreases, in contrast to manually supplying a batch of related protein sequences. UniRef90 (release November 2014) was supplied as the protein database. The SIFT score corresponds to the normalized probability that the amino acid change is tolerated. Consequently, scores near zero are more likely to be deleterious. The qualitative descriptor was supplied by Ensembl and substitutions with a score < 0.05 were considered "deleterious". **PolyPhen-2 performs prediction of functional consequences of genetic substitutions based on several sequence- and structure-based features [110]. For Ensembl, PolyPhen-2 version 2.2.2, release 405c was run following all instructions from the authors and using the UniProtKB UniRef100 (release October 2013) non-redundant protein set as the protein database and DSSP (snapshot 22 Nov 2013) and PDB (snapshot 22 Nov 2013) as the structural databases. The PolyPhen-2 score represents the probability that a substitution is damaging. Values near one predicted to more likely be damaging. The qualitative cutoffs are: 0 - 0.446: "Benign", 0.446 - 0.908: "Possibly Damaging", and 0.908 - 1.000: "Probably "Damaging".

**Table 5.** Expression quantitative trait loci (eQTLs) that were found to be linked to genetic loci associated with hearing related traits in the UK Biobank.

| Lead SNP | Chr | | Locus | | Phenotype | | Linked eQTLs | Regulated gene(s) | | Tissues | |  |
| --- | --- | --- | --- | --- | --- | --- | --- | --- | --- | --- | --- | --- |
| rs36089056 | | 1 | | 46,027,355 - 46,598,273 | | Hearing difficulty | rs6429576, rs12027345, rs11211188, rs7526532, rs12565042, rs10789475, rs9787208, rs12144263, 1:46372564 CTT C, rs11211251, rs199555086 | | *IPP, MAST2, PIK3R3* | | Thyroid, Heart Atrial Appendage, Esophagus Muscularis, Heart Left Ventricle, Whole Blood, Cells Transformed fibroblasts, Adipose Subcutaneous, Colon Sigmoid, Lung, Artery Aorta, Uterus, Artery Tibial, Nerve Tibial. | |
| rs1981809 | | 5 | | 72,838,222 - 72,995,701 | | Hearing difficulty | rs11386152, rs3822452, rs10942676, rs35211645, rs6890164 | | *UTP15, ARGHEF28* | | Nerve Tibial, Adipose Subcutaneous, Thyroid, Artery Tibial. | |
| rs13171669 | | 5 | | 148,562,829 - 148,615,946 | | Hearing difficulty | rs13171669 | | *ABLIM3* | | Cerebellum, Cerebellar Hemisphere, Hippocampus | |
| rs553448379 | | 6 | | 43,260,660 - 43,408,005 | | Hearing difficulty | rs1574430, rs2254303 | | *SLC22A7, CRIP3* | | Testis, Heart Left Ventricle | |
| rs1558804 | | 12 | | 109,788,598 - 110,042,348 | | Hearing difficulty | rs7313797, rs3742015 | | *MMAB* | | Thyroid, Colon Transverse. | |
| rs17671352 | | 17 | | 7,116,154 - 7,172,609 | | Hearing difficulty | rs929229, rs35224044, rs507506, rs739669, rs222851, rs34926505, rs2654186, rs414206, rs62059170, rs2074219, rs427784, rs2106842, 17:7165395 CG C, rs34958987, rs749977065 | | *ELP5, ACADVL, CTDNEP1, CLDN7* | | Adipose Subcutaneous, Artery Tibial, Whole Blood, Colon Sigmoid, Lung, Muscle Skeletal, Artery Aorta, Thyroid, Heart Left Ventricle, Esophagus Mucosa, Heart Atrial Appendage, Esophagus Muscularis, Testis, Stomach, Nerve Tibial, Cells Transformed fibroblasts, Adipose Visceral Omentum, Spleen. | |
| rs11152089 | | 18 | | 52,598,090 - 52,638,831 | | Hearing difficulty | rs4445975, rs1344011, rs72928899, rs199681604 | | *CCDC68* | | Muscle Skeletal, Thyroid, Heart Left Ventricle, Esophagus Muscularis. | |

The lead SNP, chromosome (Chr), locus coordinates and phenotype are presented for each locus. See the methods section for details on how eQTLs were determined to be linked to the genetic loci that were determined to be associated with hearing related traits. All eQTLs and regulated genes are presented. All tissues where eQTL regulated gene expression has been observed in the GTEx database is presented.

**Table 6. Summary of expression patterns of candidate proteins in other mammals.**

|  | Other discrete structures/cells | | Spiral Ganglion | Inner and outer hair cells | | | | Supporting cells of the membranous labyrinth | | | | | | | | | |
| --- | --- | --- | --- | --- | --- | --- | --- | --- | --- | --- | --- | --- | --- | --- | --- | --- | --- |
| Protein | Otic plate | Cochlear melano-cytes | Neuronal cells | Cuticular plates | Stereo-cilia | Nucleus | Cytosol | | Inner sulcus cells | Inter-dental cells | Border cells | Phalangeal cells | Pillar cells | Deiters’ cells | Hensen's cells | Boettcher cells | Cells of Claudius |
| EYA4* [2] |  | - | + | - | - | + | - | | + | (+) | + | + | + | + | + | - | + |
| LMX1A*^a^* | + | - | - | - | - | - | - | | - | - | - | - | - | - | - | - | - |
| PTK2/FAK^#^ |  | - | - | - | + | + | - | | - | - | - | - | + | + | + | - | - |
| UBE3B** |  |  |  |  |  |  |  | |  |  |  |  |  |  |  |  |  |
| MMP2^##^ |  | - | + | - | - | - | - | | - | - | - | - | - | - | - | - | - |
| SYNJ2*^b^* |  | - | + | - | - | - | + | | - | - | - | - | - | - | - | + | - |
| GRM5** | NA | NA | NA | NA | NA | NA | NA | | NA | NA | NA | NA | NA | NA | NA | NA | NA |
| TRIOBP |  | - | + | + | + | - | - | | - | - | - | - | - | - | - | - | - |
| LMO7 |  | - | *-* | + | - | - | - | | - | - | - | - | - | - | - | - | - |
| NOX4** | NA | NA | NA | NA | NA | NA | NA | | NA | NA | NA | NA | NA | NA | NA | NA | NA |
| MMAB** | NA | NA | NA | NA | NA | NA | NA | | NA | NA | NA | NA | NA | NA | NA | NA | NA |
| TYR*^c^* |  | *+^b^* | *-* | - | - | - | - | | - | - | - | - | - | - | - | - | - |

'+' positive protein expression detected by immunohistochemical staining. '-' expression in these structures were not reported in previous cochlear staining experiments. (+) Staining is visible in the publication's Figures, but not highlighted in the text.

*Immunohistochemical staining results in cochlea from adult marmoset *(Callithrix jacchus,* 3-6 years) [32].

**Protein expression has not been studied in other species. Therefore, data for these structures are not available (NA).

^#^Immunohistochemical staining results in cochlea from adult long-tailed chinchilla *(Chinchilla lanigera)* [111].

^##^Immunohistochemical staining results in cochlea from 5-day-old Wistar rat pups [112] .

*^a^*Immunohistochemical staining results in otic plates from male Tg (Pax2-cre)^Akg1^ mice at embryonic day E10.5 and E11.5 [113]

*^b^*Immunohistochemical staining results in cochlea from 4- and 12-week old C57BL/6 mice [44, 114].

*^c^*Immunohistochemical staining in melanocyte cell cultures from the temporal bone of 2-month-old sheep [115].

**Fig 3. Comparison of results from the current study and the recent GWAS for hearing difficulty.** **a)** P values for the genome-wide significant, and independently associated SNPs in the current study plotted against the P-values obtained by Wells *et al*. [7] Red points denote SNPs that were significant in both studies and pink points denote SNPs that were significant in our study but not in the recent GWAS. **b)** P values for the genome-wide significant, and independently associated SNPs in the GWAS by Wells *et al.* plotted against the P-values obtained in our study. Red points denote SNPs that were significant in both studies and pink points denote SNPs that were significant in the recent GWAS but not in our analyses.

**Table 7. Characteristics of tissue donors.**

| Age | Gender | PTT/SD |
| --- | --- | --- |
| 43 | Female | 50 dB (1-8kHz) |
| 51 | Male | Normal |
| 72 | Male | 50 dB (2-4kHz) |
| 67 | Female | Normal |
| 67 | Female | SD 85% |

PTT = pure tone thresholds, SD = speech discrimination.

**Table 8. Antibodies used for immunohistochemical staining.**

| Antibody | Type | Host | Catalog no. | RRID | Producer |
| --- | --- | --- | --- | --- | --- |
| LMO7 | Polyclonal | Rabbit | PA5-54281 | AB_2643409 | Invitrogen. |
| NOX4 | Polyclonal | Rabbit | PA5-85479 | AB_2792619 | Invitrogen |
| TRIOBP | Polyclonal | Rabbit | NBP2-88482 | not available | Novus |
| MMP2/2C1 | Monoclonal | Mouse | MA116640 | AB_568620 | Invitrogen |
| SYNJ2 | Polyclonal | Rabbit | PA5-56784 | AB_2648122 | Invitrogen |
| UBE3B | Polyclonal | Rabbit | NBP1-92559 | AB_11005223 | Novus |
| EYA4 | Polyclonal | Rabbit | NBP1-85548 | AB_11042949 | Novus |
| LMX1A | Polyclonal | Rabbit | NBP2-41193 | not available | Novus |
| GRM5 | Monoclonal | Mouse | MABN540 | not available | Sigma-Aldrich |
| PTK2 | Monoclonal | Mouse | 39-6500 | AB_2533427 | Invitrogen |
